# Preclinical modeling of chronic inhibition of the Parkinson’s disease associated kinase LRRK2 reveals altered function of the endolysosomal system in vivo

**DOI:** 10.1186/s13024-021-00441-8

**Published:** 2021-03-19

**Authors:** Jillian H. Kluss, Melissa Conti Mazza, Yan Li, Claudia Manzoni, Patrick A. Lewis, Mark R. Cookson, Adamantios Mamais

**Affiliations:** 1 Cell Biology and Gene Expression Section, Laboratory of Neurogenetics, National Institute on Aging, National Institutes of Health, Bethesda, MD USA; 2 School of Pharmacy, University of Reading, Whiteknights Campus, Reading, UK; 3 Proteomic Core Facility, National Institute of Neurological Disorders and Stroke, Bethesda, MD USA; 4 UCL School of Pharmacy, Brunswick Square, London, UK; 5 Royal Veterinary College, Royal College Street, London, UK; 6 Department of Neurodegenerative Diseases, UCL Queen Square Institute of Neurology, London, UK; 7 Department of Neuroscience, Center for Translational Research in Neurodegenerative Disease, Norman Fixel Institute for Neurological Diseases, University of Florida College of Medicine, Gainesville, Florida USA

## Abstract

The most common mutation in the Leucine-rich repeat kinase 2 gene (LRRK2), G2019S, causes familial Parkinson’s Disease (PD) and renders the encoded protein kinase hyperactive. While targeting LRRK2 activity is currently being tested in clinical trials as a therapeutic avenue for PD, to date, the molecular effects of chronic LRRK2 inhibition have not yet been examined in vivo. We evaluated the utility of newly available phospho-antibodies for Rab substrates and LRRK2 autophosphorylation to examine the pharmacodynamic response to treatment with the potent and specific LRRK2 inhibitor, MLi-2, in brain and peripheral tissue in G2019S LRRK2 knock-in mice. We report higher sensitivity of LRRK2 autophosphorylation to MLi-2 treatment and slower recovery in washout conditions compared to Rab GTPases phosphorylation, and we identify pS106 Rab12 as a robust readout of downstream LRRK2 activity across tissues. The downstream effects of long-term chronic LRRK2 inhibition in vivo were evaluated in G2019S LRRK2 knock-in mice by phospho- and total proteomic analyses following an in-diet administration of MLi-2 for 10 weeks. We observed significant alterations in endolysosomal and trafficking pathways in the kidney that were sensitive to MLi-2 treatment and were validated biochemically. Furthermore, a subtle but distinct biochemical signature affecting mitochondrial proteins was observed in brain tissue in the same animals that, again, was reverted by kinase inhibition. Proteomic analysis in the lung did not detect any major pathway of dysregulation that would be indicative of pulmonary impairment. This is the first study to examine the molecular underpinnings of chronic LRRK2 inhibition in a preclinical in vivo PD model and highlights cellular processes that may be influenced by therapeutic strategies aimed at restoring LRRK2 physiological activity in PD patients.

**Supplementary Information:**

The online version contains supplementary material available at 10.1186/s13024-021-00441-8.

## Background

Mutations in *leucine-rich repeat kinase 2* (*LRRK2*) are a known genetic cause of familial Parkinson’s disease (PD) [1–3];. Non-coding variation at the *LRRK2* locus has also been identified as a risk factor for sporadic PD, suggesting that both disease forms share common pathological mechanisms [4, 5]. The G2019S mutation, which lies within the kinase domain of LRRK2, is the most common mutation found in familial PD cases, as well as in 1–5% of apparently sporadic PD patients [6]. This mutation directly increases kinase activity while other mutations likely have convergent cellular effects, albeit through varying mechanisms [4]. Therefore, it is thought that targeting LRRK2 therapeutically may be advantageous in both familial and sporadic PD [7, 8].

A number of pharmacological tools that inhibit LRRK2 kinase activity in the CNS have been developed and characterized [9]. Some kinase inhibitors have been shown to reduce cytotoxicity associated with LRRK2 mutations in PD-relevant cell and animal models [10–12], supporting the hypothesis that LRRK2 inhibition may be efficacious for PD. One such LRRK2-specific inhibitor, MLi-2, has been found to be ~300x more selective for LRRK2 over other kinases and can readily cross the blood-brain barrier [13] demonstrating that it is possible to generate tool compounds that have clinical potential for therapeutic intervention in PD. However, as LRRK2 is expressed endogenously in many tissues and kinase inhibition is predicted to affect both mutant and wild-type LRRK2, whether such inhibitors would be safe to use clinically is uncertain. Preclinical studies have reported macroscopic changes in vivo that include morphological changes in lung from nonhuman primates, and kidney tissue from rats treated with specific LRRK2 inhibitors that are reversible after termination of treatment [14–16]. Importantly, some of these effects overlap with those seen in Lrrk2 knockout mice [17] demonstrating that they result from on-target effects of inhibitors on LRRK2 itself rather than resulting from inhibition of other kinases.

Here, we aimed to mimic a likely clinical use of LRRK2 inhibitor to understand the relationship between level of inhibition and effects on biochemistry of target tissues including brain and peripheral organs that express high levels of LRRK2. We first evaluated the utility of measuring autophosphorylation of LRRK2 vs downstream substrate Rab proteins as readouts of LRRK2 kinase activity in vivo across a series of acute and chronic dosing paradigms in kinase hyperactive G2019S LRRK2 knock-in mouse model. Secondly, we explore the molecular effects of long-term chronic LRRK2 inhibition by unbiased total and phospho-proteomics on brain, kidney, and lung tissue from G2019S LRRK2 mice following 60 mg/kg/day in-diet dosing of MLi-2 for 10 weeks. Based on these data, we report molecular pathways that are affected by chronic inhibition of LRRK2 in a clinically relevant paradigm. Importantly, we show that the effects of LRRK2 inhibition vary across tissues, which may be particularly relevant to selection of biomarkers for clinical trials where brain target engagement is inferred from peripheral tissue events.

## Methods

### Animals

All animal procedures were performed in accordance with a protocol approved by the Institutional Animal Care and Use Committee of the National Institute on Aging, NIH.

Wildtype, G2019S LRRK2 knock-in (KI), and LRRK2 knockout (KO) male and female mice raised on a C57Bl/6 background (3–8 months in age) were bred in-house on a 12-h day/night cycle for the following experiments. All mice were supplied with Rodent NIH-07 diet and water ad libitum.

### Acute MLi-2 dosing

*Dose response:* 28 homozygous G2019S KI C57BL/6 J mice (5–8 months old) were randomized for treatment using the sample function in R, followed by matching for sex across groups. Mice were given an acute dose of vehicle [40% (w/v) Hydroxypropyl-β-Cyclodextran] or MLi-2 (at 1, 3, 10, 30, 60, or 90 mg/kg dissolved in vehicle) via oral gavage and euthanized 1 h after treatment. Based on our previous data on S1292 dephosphorylation in G2019S LRRK2 brain following acute MLi-2 treatment [18], *N* = 4 was used in this study for all acute MLi-2 experiments, as it was estimated at 90% power to detect a difference of effect size 6 at alpha = 0.05.

*Time course:* 28 homozygous G2019S KI C57BL/6 J mice (4–7 months old) were randomized for treatment using the sample function in R, followed by matching for sex across groups. Mice were given an acute dose of vehicle [40% (w/v) Hydroxypropyl-β-Cyclodextran] at time point zero, or MLi-2 (10 mg/kg dissolved in vehicle) via oral gavage and euthanized after time point 0.5, 1, 3, 12, 24, or 72 h post dose.

### Chronic in-diet dosing

To determine the appropriate concentration of MLi-2 necessary to achieve S1292 phosphorylation levels that are comparable to the brain tissue levels in a wildtype animal, homozygous G2019S KI mice were given customized Rodent NIH-07 chow from Research Diets, either untreated or supplemented with various doses of MLi-2 (10, 30, or 60 mg/kg/day) for 10 days along with an untreated wildtype group, and S1292 phosphorylation signal was measured via Western blot (*N* = 3 mice were used per treatment group). Mice and chow were weighed daily to assess estimated doses of MLi-2 received each day. No difference was observed in food intake between mice receiving MLi-2 treated chow and mice receiving untreated chow. All mice were given chow and water ad libitum. After 10 days, mice were euthanized 2-3 h post start of their light cycle.

*Ten day (short-term) and 10 week (long-term) chronic cohorts:* Three-month old littermates from wildtype, homozygous G2019S KI, and homozygous LRRK2 KO litters were randomized into short-term (10 days) and long-term (10 weeks) chronic treatment groups: wildtype, G2019S KI, and LRRK2 KO groups received untreated chow, and a G2019S KI group received chow supplemented with 60 mg/kg of MLi-2 per day. Both male and female mice were represented equally in all treatment groups (*N* = 6 mice were used per treatment group). All mice were given chow and water ad libitum. After 10 days, or 10 weeks, mice were euthanized 2-3 h post start of their light cycle.

### Statistical analyses

Statistical analyses for each experiment can be found within each respective figure legend. Briefly, one-way ANOVA or two-way ANOVA with Tukey’s post hoc-test were used which were performed in GraphPad Prism v8.2.0 (GraphPad Software, San Diego, CA). If not otherwise stated, comparisons were considered statistically significant where *p* < 0.05. *, *p* < 0.05; **, *p* < 0.01; ***, *p* < 0.001; ****, *p* < 0.0001.

### Immunoblotting

Tissues were homogenized at 20% w/v in 1x Cell Signaling Lysis Buffer (#9803S) with 1x protease and phosphatase inhibitors (Thermofisher; #1861279 and #78427, respectively) and left on ice for 30 min to lyse. Homogenates were spun at 20,000 g for 10 min at 4 degrees Celsius and pelleted debris were removed. Samples were supplemented with NuPage LDS sample buffer 4x (#NP0008), boiled for 5 min at 95 degrees Celsius and run on a Bio-Rad Criterion™ TGX™ polyacrylamide gel (#5671095) at 200 V for 37 min. Gels were transferred to nitrocellulose (#1704159) on a Bio-Rad Trans-Blot Turbo™ transfer system at 20 V for 10 min. A normalizer sample was added to the end of each blot to account for day-to-day variability in protein transfer and antibody incubation. This stock was prepared prior to beginning this study and single-use aliquots were prepared in loading buffer and stored at -80C until use to ensure no loss of phosphorylation nor protein degradation. The nitrocellulose was blocked for 1 h in 50% TBS (20 mM Tris, 0.5 M NaCl, pH 7.5), 50% Odyssey blocking buffer (Li-Cor; 927–40,000) and incubated overnight with primary antibodies in 50% TBS-T (20 mM Tris, 0.5 M NaCl, pH 7.5, 0.1% Tween 20), 50% Odyssey blocking buffer at 4 degrees Celsius. All antibody concentrations used in this study can be found in Table S1. Following 3 × 5 min washes with TBS-T, the nitrocellulose was incubated at RT with secondary antibodies for 1 h, washed 3 × 5 min and scanned at the Li-Cor platform.

All primary antibodies and working dilutions can be found in Table S1. Secondary antibodies were used at 1:10,000 dilution: IRDyes 800CW Goat anti-Rabbit IgG (LiCor; #926–32,211) and 680RD Goat anti-Mouse IgG (LiCor; 926–68,070). All blots presented in each figure panel were derived from the same experiment and were processed in parallel. Raw densitometric signal was normalized to a common normalizer sample that was run on every blot.

### Tissue preparation for proteomics and phosphoproteomics

One brain hemisphere, one half of a kidney, and one whole lung from each mouse in the short-term and long-term chronic experiments were processed and submitted for proteomics and phosphoproteomics analysis using TMT quantitation without and with TiO_2_/iMAC enrichment. Briefly, tissues were homogenized in 15% w/v lysis buffer (0.5 mM HEPES, pH 7.4, 225 mM mannitol, 50 mM sucrose, 1 mM EDTA, 1x protease and phosphatase inhibitors (Thermofisher; #1861279 and #78427, respectively), and 2% CHAPS) followed by incubation on ice for 30 min, with periodic vortexing, to lyse. The homogenates were then spun down at 20,000 g at 4 degrees Celsius for 10 min to pellet cellular debris. A protein assay was performed on supernatants to determine protein concentration, and 270 μg of each sample were submitted for mass spectrometry. Additionally, 30 μg of each sample were pooled together to allow for normalization between runs during analysis.

### Phosphoproteomics and bioinformatics analysis

Proteins were alkylated with NEM, digested with trypsin and labeled with TMTpro reagents. The brain and kidney samples were treated with the same method. Forty eight brain/kidney samples and 4 pooled samples were separated into 4 sets of TMT experiments. Each set contains 12 samples and one pooled sample. Twenty-four whole lung samples and 2 pooled samples were separated into 2 sets of TMT experiments. Each set contains 12 samples and one pooled sample. The pooled sample in each set was labeled with TMTpro-126 reagent, while the distribution and labeling of 12 samples were randomized (Data File S1). After the labeling and quenching, samples were combined together. 95% of the combined sample was used for phosphopeptide enrichment using TiO2 method followed by iMAC method. 5% of the combined sample was fractionated using Pierce high pH reverse phase cartridge, and 8 fractions were collected for each set. LC-MS/MS data acquisition were performed on a Thermo Scientific Orbitrap Lumos mass spectrometer which was coupled to a Thermo Scientific Ultimate 3000 HPLC. Peptides were separated on an ES802 nano-column over 136 min at a flow rate of 300 n/min. TMT MS2 method was used. Both MS1 and MS2 scans were performed in orbitrap. The resolution for MS1 and MS2 scans were 120 K and 50 K, respectively. Peptides were fragmented using the HCD method with collision energy fixed at 32%. The precursor isolation window is 1.5 Da.

Proteome Discoverer 2.4 was used for database search and TMT quantitation. Ratios of raw abundance values of each sample over the pooled sample were generated and analyzed in R using the limma package (version 4.0, empirical Bayes method) [19]. Briefly, the proteomics datasets from four MS runs (or two for the lung samples) were combined and only hits that were detected in at least 4 out of 6 mice were kept, while the missing 1–2 values were imputed. These phospho-peptide and total protein datasets were merged and matched for accession number and the ratio of phospho/total abundance was generated for phosphopeptide significant hit detection. Principal component analysis was conducted for these datasets which revealed a modest batch effect that was corrected computationally. Statistical analyses were conducted, namely moderated t-tests between pairs were visualized as volcano plots [20], and z scores were calculated for heatmaps. Heatmaps were curated from statistically significant hits selected based on relevance to neurodegeneration and reported functions on cellular pathways affected in Parkinson’s Disease. Proteins were considered significant hits with an adjusted *p*-value < 0.05, and for kidneys a fold change > 1.4. Functional enrichment analysis of the significant hits was performed using the R package gProfiler2 (precision cutoffs of adjusted *p*-values were < 1e-6 and < 1e-20 for kidney and brain hits, respectively, and term size < 1000 was kept [21]. All hits from the lungs did not return any significant terms within these parameters. Enrichment term networks were visualized using Cytoscape 3.7 [22].

Additionally, we generated a human interactome of LRRK2 and compared it with the significant hits found in kidney total and phospho- proteomics (Fig. S7) using the PINOT searching tool (http://www.reading.ac.uk/bioinf/PINOT/PINOT_form.html) which collates published, experimentally validated protein-protein interaction (PPI) data from 7 different databases (bhf-ucl, BioGRID, InnateDB, IntAct, MBInfo, MINT and UniProt), merged the annotations and performed quality control providing a final score indicative of reproducibility [23]. The PPIs of LRRK2 with a final score > 2 were kept as first layer interactors (i.e. reported in literature as able to directly bind to LRRK2). The first layer interactors of LRRK2 (220 proteins) were used as input in PINOT to download PPIs able to bind directly to the first layer (i.e. second layer interactors). The second layer interactors are proteins that are able to connect to LRRK2 via their direct connection with a protein within the first layer (Data File S2). Scripts were run in R (3.6.2) and networks were drawn using Cytoscape 3.5, mouse to human protein ID conversion was performed using g:Profiler [21].

## Results

### Relative sensitivity of LRRK2 autophosphorylation and Rab GTPase phosphorylation to MLi-2 inhibition in vivo

Our aim in this series of studies was to mimic a likely clinical scenario where mutant LRRK2 is inhibited to levels similar to that seen with wild type protein, thus nullifying the potential toxic effects of enhanced pathogenic kinase activity. In order to be able to model this situation, we first needed to identify reliable markers of LRRK2 activity in vivo and understand the relationship between peripheral organ and brain target engagement. To this end, we compared the response of phosphorylation of LRRK2 itself and downstream Rab substrates to MLi-2 in vivo using two acute treatment paradigms*.*

**Fig. 1:**
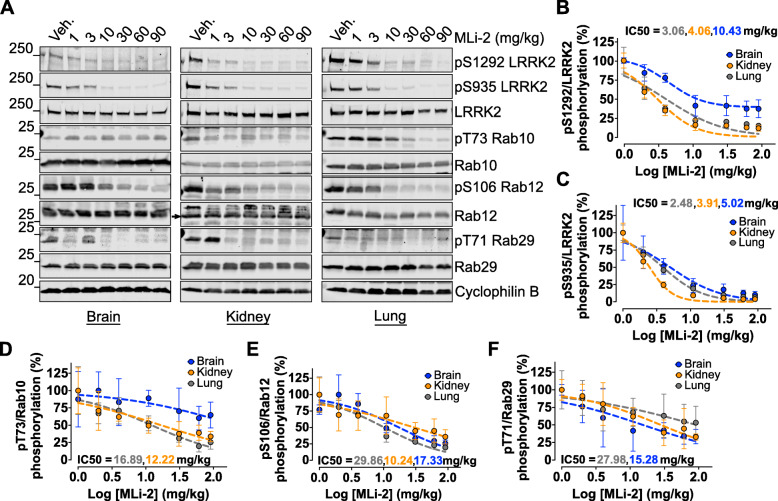
Dose response of acute MLi-2 across brain and peripheral tissues. G2019S LRRK2 knock-in mice were given a single MLi-2 dose via oral gavage and sacrificed 1 h later. Brain, kidney, and lung homogenates were analyzed by western blot (*N* = 4 per group; 60 μg of protein were loaded per well). **a, b, c** Maximal LRRK2 inhibition was achieved at 10 mg/kg MLi-2 and plateaued with increasing MLi-2 amounts as seen in S1292 and S935 phosphorylation readouts across all tissues. **d** pT73 Rab10 showed variability in brain but significant dephosphorylation in peripheral tissues with treatment. **e** pS106 Rab12 showed a robust dose-dependent dephosphorylation across all tissues. **f** pT71 Rab29 showed variability but a trend of decrease across all tissues with treatment that reached maximal inhibition at the 30-60 mg/kg points. All dose response curves of the phospho-sites are shown in **b**-**f** and represent percentage over vehicle controls of phospho-levels normalized to total protein levels. IC50 values of Rab GTPases are generally higher and more variable across tissues compared to LRRK2 phosphorylation (IC50s were not reported when 50% reduction was not reached within the dose-response tested)

First, we investigated the dose responsiveness of LRRK2 autophosphorylation and phosphorylation of Rab GTPases to acute kinase inhibition in vivo. G2019S KI mice were given an acute dose of MLi-2 at 1, 3, 10, 30, 60, or 90 mg/kg or vehicle via oral gavage and sacrificed 1 h post dose. We observed significant pS1292 dephosphorylation starting at the lowest dose of 1 mg/kg in kidney and lung (Fig. 1a, b). Maximal dephosphorylation was achieved in the brain, kidney and lung at 10 mg/kg for both pS1292 and pS935 (Fig. 1a, b, c). Maximal S1292 dephosphorylation in the brain did not exceed 60% signal decrease with increasing MLi-2 concentrations, suggesting that maximal inhibition was achieved. The S935 phospho-site had lower IC50 compared to pS1292 in the brain, suggesting that this marker is more sensitive to LRRK2 inhibition in vivo*.*

Phosphorylated Rab GTPases showed more variable responses to MLi-2 across tissues. The T73 Rab10 phospho-site responded significantly to MLi-2 treatment in peripheral tissues but not in the brain (Fig. 1a, d). Phospho- S106 Rab12 showed a robust response to MLi-2 in both brain and peripheral tissues with maximal dephosphorylation at 30-60 mg/kg (Fig. 1a, e; antibody validation experiments are shown in Figs. S2 and S3). The T71 Rab29 phospho-site responded with treatment in all tissues but with higher variability compared to Rab12, especially at lower doses (Fig. 1a, f). At 10 mg/kg, we saw ~ 50% decrease in Rab10, Rab12 and Rab29 phosphorylation in the periphery while higher doses retained ~ 30% residual phosphorylation signal. These results demonstrate that 10 mg/kg of MLi-2 is adequate to acutely inhibit LRRK2 autophosphorylation in brain and peripheral tissues. Based on these results, we selected a dose of 10 mg/kg MLi-2 for subsequent time course analyses and washout experiments.

### Time course of acute MLi-2 administration reveals that phosphorylation of Rab GTPases recovers faster than LRRK2 phosphorylation

To further discriminate how LRRK2 and Rab phosphorylation events vary between brain and peripheral tissues, we compared time courses and recovery after washout of inhibitor application. An acute dose of MLi-2 at 10 mg/kg was administered via oral gavage to G2019S KI mice and sacrificed at 0.5, 1, 3, 12, 24, or 72 h post dose. A time point of 0 h was included where animals were given an equivalent dose of vehicle and euthanized immediately thereafter to measure baseline phosphorylation.

**Fig. 2:**
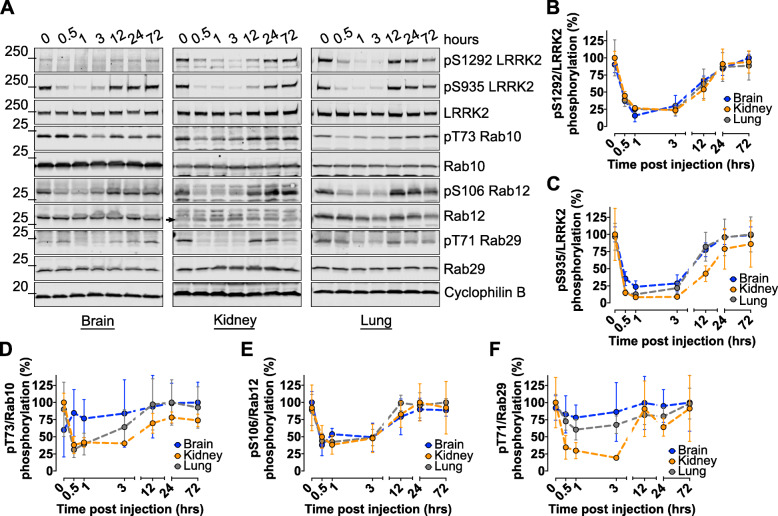
Time-course of target inhibition and rephosphorylation of LRRK2 and Rab GTPase substrates across brain and peripheral tissues. G2019S LRRK2 knock-in mice were given a single 10 mg/kg MLi-2 dose via oral gavage and sacrificed at various time points. Brain, kidney, and lung homogenates were analyzed by western blot (*n* = 4 per group; 60 μg of protein were loaded per well) **a**. Maximal LRRK2 inhibition was achieved at 1 h post dose as shown by S1292 and S935 dephosphorylation while complete rephosphorylation was achieved by 24 h **b**, **c**. Rab10 was dephosphorylated by 1 h post-dosing in kidney and lung but not in the brain that showed variability in phosphorylated levels and did not respond to treatment **a**, **d**. Rab12 showed robust dephosphorylation by 1 h in brain and periphery **a**, **e**. Rab29 responded with maximal dephosphorylation in brain and lung by 1 h while kidney showed a slower but more significant response **a, f**. All three Rab GTPases recovered their phosphorylation levels by 12 h post-dose. These results demonstrate that Rab GTpases are dephosphorylated within the first hour following LRRK2 inhibition and rephosphorylate quicker than LRRK2 autophosphorylation recovery following wash-out conditions (~ 12 h vs ~ 24 h)

Phospho- S1292 and S935 LRRK2 showed rapid dephosphorylation at 0.5 h, with maximal dephosphorylation achieved at 1 h post dose across all tissues (Fig. 2a - c). The dephosphorylation patterns of both phospho-sites tightly correlated between brain, kidney, and lung tissues. Interestingly, maximal dephosphorylation for all pRabs was seen at 1 h in all tissues. However, these sites recovered more rapidly than pLRRK2 (~ 12 h versus 24 h; Fig. 2a, d - f). Measures of T73 Rab10 signal in brain tissue did not show MLi-2-dependent dephosphorylation compared to peripheral tissues (Fig. 2a, d). Dephosphorylation of T71 Rab29 was achieved in all three tissues to varying degrees, with kidneys showing the strongest response to MLi-2, reaching ~ 75% dephosphorylation compared to 20–30% seen in brain and lung tissue (Fig. 2a, f). pS106 Rab12 mimicked pLRRK2 most closely in that all tissues showed similar dephosphorylation patterns over time and reached a maximum of 60% dephosphorylation (Fig. 2a, e). It was also noted that Rab12 phosphorylation showed the least variability across mice compared to Rab10 and Rab29. These data suggest that Rab GTPases are dephosphorylated within the first hour after LRRK2 inhibition in a similar fashion to LRRK2 dephosphorylation, with Rab12 performing similarly to pLRRK2 in the brain and periphery. In contrast, the kinetics of Rab GTPase re-phosphorylation show a quicker recovery (~ 12 h) compared to LRRK2 (~ 24 h).

### In-diet MLi-2 administration can diminish G2019S-dependent hyperphosphorylation to wild type levels

To evaluate the molecular effects of chronic LRRK2 inhibition, we first conducted a dose response experiment of MLi-2 in diet. With clinical relevance in mind, our aim was not to fully inactivate LRRK2, as inferred from a complete dephosphorylation of S1292, but to ameliorate the hyperphosphorylation to a range observed in wildtype animals.

**Fig. 3:**
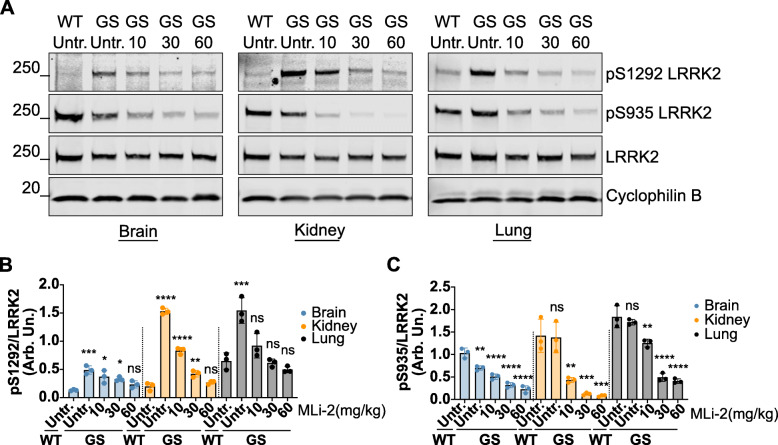
Assessment of MLi-2 in-diet dosing and LRRK2 inhibition. G2019S LRRK2 knock-in mice were fed chow containing different concentrations of MLi-2 to reach 10, 30, or 60 mg/kg/day dosing compared to G2019S knock-in and WT LRRK2 cohorts that were fed control chow. After 10-days, animals were sacrificed 2–3 h post start of their light cycle. Western blots of brain, kidney and lung tissue samples are shown. **a, b** Across all tissues, a significant increase in S1292 phosphorylation is seen in G2019S mice compared to WT animals which is ameliorated with increasing MLi-2 doses reaching WT levels at 60 mg/kg/day. **c** A dose-response was seen in S935 dephosphorylation that plateaued at 30 mg/kg/day. Quantitation of phosphorylation levels is calculated as S1292 and S935 levels over total LRRK2 (**b** and **c**: one-way ANOVA with Tukey’s post hoc, ****P < 0.0001, ***P < 0.0002, **P < 0.0021, *P < 0.0332; *n* = 3; SD bars shown; **b** *F* (14, 30) = 52.27, **c** *F* (14, 30) = 56.58)

G2019S KI mice were fed a customized rodent chow supplemented with MLi-2 to achieve 10, 30, or 60 mg/kg/day dosing. For reference, we included wildtype and G2019S KI mice that were fed control chow for 10 days. In the treated animals, we observed that 60 mg/kg/day diminished S1292 phosphorylation to wildtype levels in brain and kidney, whereas a 10 mg/kg/day dose was sufficient to decrease phosphorylation to wildtype levels in lung tissue (Fig. 3a, b). This suggests some peripheral tissues with enrichment of LRRK2 may be more sensitive to drug-induced inhibition. Increasing doses of MLi-2 show a dose response in pS935 LRRK2 (Fig. 3a, c). These results confirm a dose of 60 mg/kg/day is sufficient to inhibit G2019S LRRK2 to wild type levels in vivo across tissues.

### Chronic MLi-2 treatment in G2019S KI mice results in sustained LRRK2 and Rab12 dephosphorylation

**Fig. 4:**
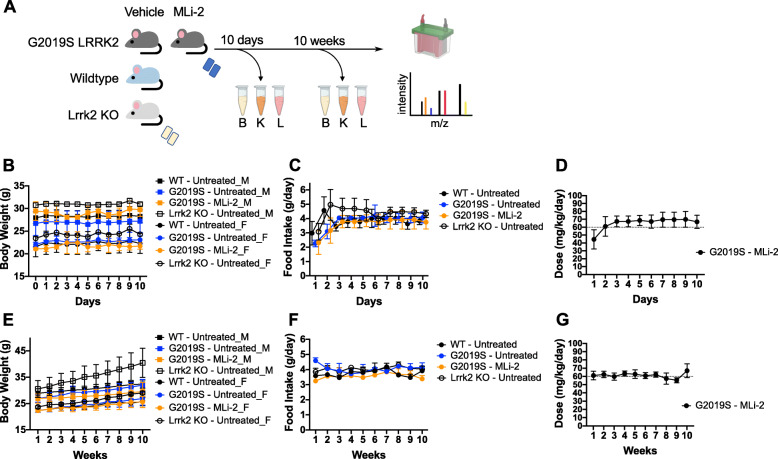
Experimental design and dosing paradigm of the chronic in vivo MLi-2 proteomics experiment. **a** Schematic of experimental design for the chronic MLi-2 proteomics experiment. **b** 10-Day cohort animals observed no weight changes (male and female body weight trends are shown separated within genotypes as square and circle points, respectively) and consumed ~ 4 g of chow per day **c**. Daily dose of MLi-2 was calculated by body weight and food intake and showed that animals on average received ~ 65 mg/kg/day of drug **d**. **e** 10-Week cohort animals grew 3-8 g in body weight over the course of 10 weeks (shown as % weight change), with an average food intake of 4 g **f** and hit the 60 mg/kg/day mark each week **g**

To extend these results into a chronic timescale, G2019S KI mice were given customized chow supplemented with MLi-2 to reach a 60 mg/kg/day dose for 10 days or 10 weeks. Control groups of wildtype, G2019S KI, and LRRK2 KO mice receiving untreated chow were included for reference of baseline phosphorylation patterns. For the purpose of this experiment, we refer to the 10-day cohort as ‘short-term’ and the 10-week cohort as ‘long-term’ treatment groups. The schematic in Fig. 4a depicts the design of this experiment, in which brain, kidney, and lung tissues were collected and processed for Western blot analyses and additionally prepped for total and phospho-proteomics. Body weight and estimated food intake for each mouse was recorded daily to determine the daily dose of MLi-2 each mouse received (Fig. 4b - g). Both short- and long-term cohorts received the appropriate dose of MLi-2 and had comparable chow intake of ~ 4 g, while weight increase was observed only in the 10-week cohort, particularly in the LRRK2 KO animals.

**Fig. 5:**
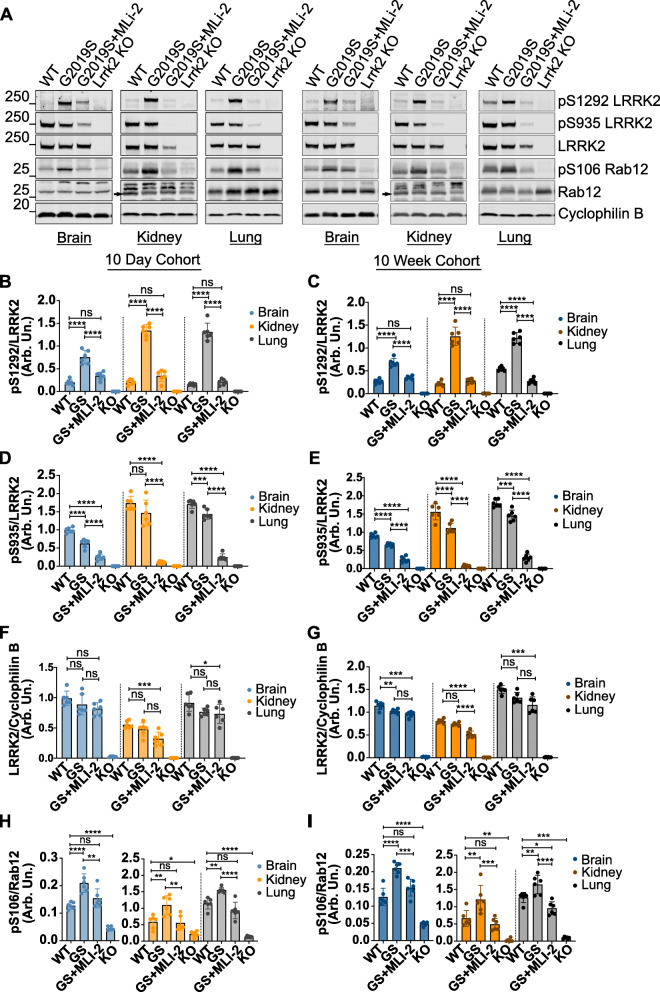
LRRK2 and S106 Rab12 phosphorylation following chronic MLi-2 treatment. WT, G2019S LRRK2 KI, and LRRK2 KO mice were fed vehicle or MLi-2-containing chow over the course of 10 days or 10 weeks **a**. LRRK2 inhibition is maintained between the short-term (10-day) and long-term (10-week) chronic treatment as shown by decrease in pS935 levels **d**, **e** and S1292 LRRK2 dephosphorylation **b, c** to levels comparable to WT controls. A decrease in total LRRK2 levels is observed in peripheral tissues with chronic treatment in by 10 days **f** and is exacerbated in the 10-week treatment **g**. S106 Rab12 phosphorylation levels show a significant decrease with treatment to levels comparable to WT tissue **h**, **i**. One-way ANOVA with Tukey’s post hoc; *n* = 6; ****P < 0.0001, ***P < 0.0002, **P < 0.005, *P < 0.05; **b** *F* (11, 60) = 146.8, **c** *F* (11, 60) = 178.7, **d** *F* (11, 60) = 158.0, **e** *F* (11, 60) = 265.9, **f** *F* (11, 60) = 75.97, **g** *F* (11, 60) = 271.5, **h** F = (11, 60) = 65.78, **i** F (11, 60) = 59.67

In both short-term and long-term cohorts, pS1292 levels of G2019S KI mice treated with MLi-2 were significantly decreased to levels comparable to wildtype mice (Fig. 5a - c). In the long-term cohort, mice treated with MLi-2 showed further dephosphorylation compared to wildtype levels in lung (Fig. 5c). S106 Rab12 showed significant dephosphorylation consistent throughout tissues, similar to the response of LRRK2 dephosphorylation (Fig. 5a, h - i), proving pRab12 is a reliable readout of LRRK2 activity and inhibition in this model. Additionally, phosphorylation of S935 LRRK2 was significantly reduced in all tissues compared to both G2019S and wildtype untreated animals in both cohorts as expected (Fig. 5a, d - e). In contrast, and similar to the tissue and dose-specific responses to acute inhibition, Rab10 and Rab29 did not respond consistently to LRRK2 inhibition in either 10-day or 10-week groups (Fig. S4 A-F). To test for covariates of Rab10 phosphorylation, we ran Pearson’s correlation coefficient on all dosing experiments in this study, testing for batch of tissue processing, gender, dose, age, treatment, and timepoints and found no contributing factor for the variation in phosphorylation (Fig. S4G). Furthermore, total LRRK2 levels in the 10-day MLi-2 cohort were comparable to their untreated G2019S counterparts in all tissues, whereas in the 10-week groups, there was a significant decrease in LRRK2 levels in kidney with treatment (Fig. 5a, f - g). This suggests chronic inactivation of LRRK2 leads to accelerated protein degradation, consistent with previous in vivo and in vitro studies using MLi-2 and other LRRK2 inhibitors [13, 24].

### Unbiased proteomics reveal both therapeutic and dysregulatory effects in endolysosomal, trafficking, and mitochondrial pathways with chronic LRRK2 inhibition in mice

The above studies identified a chronic dosing regimen in which amelioration of the hyperphosphorylation of the S1292 autophosphorylation LRRK2 site and pS106 Rab12 in G2019S KI mice can be achieved to levels seen with wildtype LRRK2 at the endogenous level in vivo. We next used a series of proteomics approaches to determine, in an unbiased manner, what the consequences of this treatment might be to tissue proteome.

**Fig. 6:**
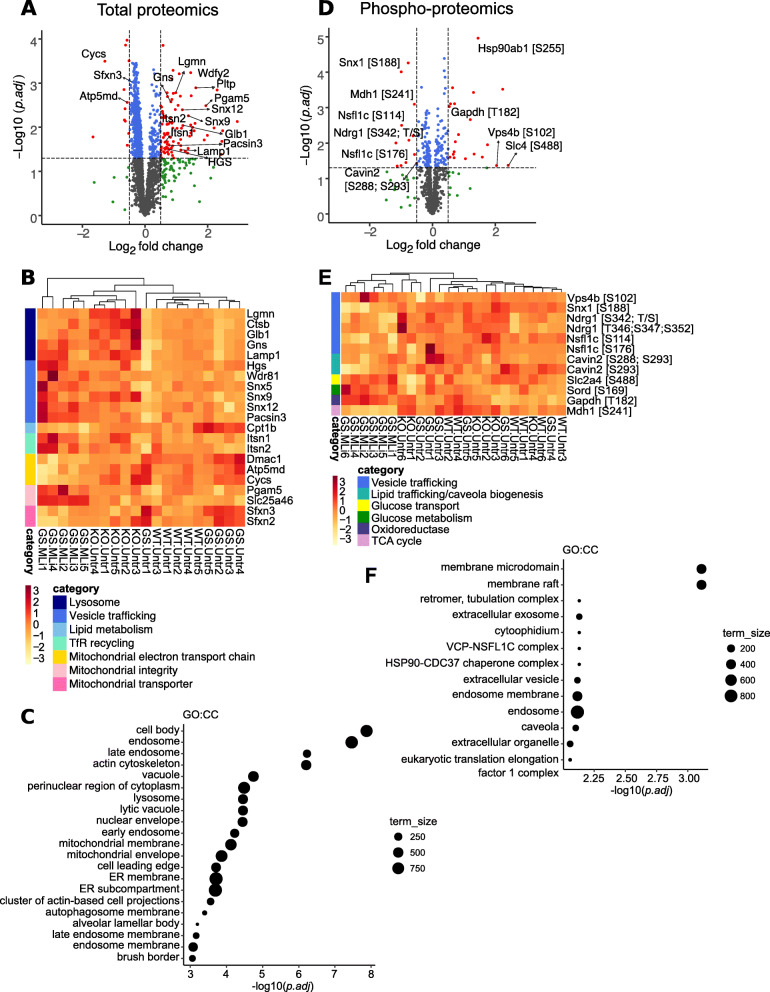
**Proteomic analysis of changes in kidney between G2019S LRRK2 MLi-2 and G2019S vehicle 10-week treated mouse cohorts. a** Volcano plot showing changes in protein levels following chronic treatment, depicted as log2 fold changes (*x*-axis) versus the −log10 of adjusted *p*-values (*y*-axis) for each protein. A number of endolysosomal proteins were upregulated, including legumain and other lysosomal proteases in kidneys of G2019S LRRK2 KI mice that received chronic MLi-2 treatment for 10 weeks, compared to vehicle G2019S KI controls. **b** Heat maps of selected significant hits across the WT, G2019S KI and LRRK2 KO vehicle cohorts as well as the G2019S KI chronic MLi-2 cohort, highlighting proteins involved in vesicular trafficking, lipid metabolism, transferrin recycling and mitochondrial homeostasis. Z score is used for scaling. Using unsupervised hierarchical clustering we show that the G2019S MLi-2 and LRRK2 KO mice cluster together, suggesting that chronic LRRK2 inhibition mimics a LRRK2 KO effect in kidney. **c** Bubble plot showing GO:CC term enrichment in the significant total protein hits highlights enrichment for endolysosomal processes and compartments. **d** Volcano plot showing changes in phospho-proteins in G2019S MLi-2 treated vs G2019S untreated mice, highlighting dysregulation of proteins involved in trafficking, glucose transport and metabolism, and the TCA cycle **e**. **f** Bubble plot showing GO:CC term enrichment in the significant phospho-peptide hits highlights enrichment for endolysosomal processes as well as kidney-specific functional processes. Volcano plots key: grey = non-significant, blue = < 0.05 *p*-value, green = > 1.4x log fold change, red = < 0.05 p-value and > 1.4x log fold change

Proteomic analysis revealed 115 total proteins and 34 phospho-proteins that were differentially expressed in the kidney between chronic MLi-2-treated and untreated G2019S LRRK2 mice (false-discovery rate (FDR) adjusted *p* < 0.05; fold change (FC) > 1.4); (Fig. 6a, b). Among the top differentially abundant proteins, there was a strong enrichment for endolysosomal, trafficking and mitochondrial proteins (Fig. 6a, b). Multiple lysosomal proteins showed differential abundance, including cathepsin B (Ctsb), legumain (Lgmn), galactosidase beta 1 (Glb1), Lysosomal-associated membrane protein 1 (Lamp1), and N-acetylglucosamine-6-sulfatase (Gns). In addition, a number of proteins involved in vesicular trafficking, lipid metabolism, iron uptake and mitochondrial function were also significantly altered in kidneys of chronically treated animals. Hierarchical clustering of differential proteins in the G2019S MLi-2 treated group showed most similarity to the LRRK2 KO animals (Fig. 6c), suggesting that chronic inhibition of LRRK2 may mimic features of an absence of LRRK2 in the periphery. Among the significant phosphoprotein hits, additional trafficking and mitochondrial proteins were identified, including sorting nexin 1 (pS188 Snx1) and vacuolar sorting protein 4b (pS102 Vps4b) (Fig. 6d). Analysis using Gene Ontology databases of significant hits from total and phospho-proteins showed enrichment of the endolysosomal system as well as mitochondrial membrane (Fig. 6e, f).

Additionally, we analyzed the human homologs of total and phospho-protein hits in silico to identify a LRRK2 protein-protein interactome using PINOT [23]. We converted the 115 total proteins and 34 phospho-proteins that were differentially expressed in the kidney between chronic MLi-2-treated and untreated G2019S LRRK2 mice to their human orthologues and identified 76 matches within the LRRK2 interactome. Five of the matching proteins (AHCYL1, EEF2, HSP90AA1, HSP90AB1 and RANBP2) were present in the first layer while 71 in the second layer of LRRK2 interactions. The difference between the average random result from 100,000 simulated experiments (40 matches) and the real result (76 matches) was highly significant (*p* = 2.78*10^− 11^). The matching proteins were extracted from the LRRK2 interactome and their connectivity with LRRK2 visualized (Fig. S7). This high degree of connectivity suggests that these proteins are in fact related to LRRK2 biology rather than an effect of MLi-2 treatment itself.

**Fig. 7:**
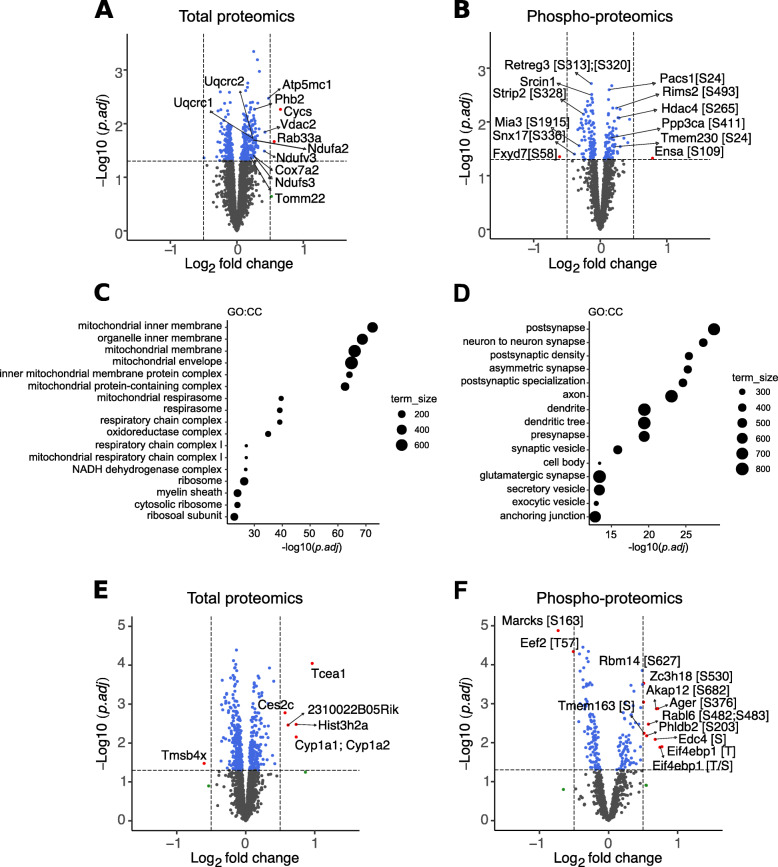
Proteomic analysis of changes in brain and lung between G2019S LRRK2 MLi-2 and G2019S vehicle 10-week treated mouse cohorts**.** A volcano plot from total proteomics of whole brain tissue comparing the G2019S chronic MLi-2 cohort versus G2019S KI vehicle controls **a**. Modest upregulation was observed in mitochondrial proteins with treatment, specifically proteins of the electron transport chain **a**. Heatmap visualizes changes in phosphopeptides with treatment highlighted proteins involved in vesicular trafficking and postsynaptic regulation on a z score scale **b**. GO:CC term enrichment bubble plots were generated from the significant hits of the total and phospho-proteomics showing high enrichment for mitochondrial compartments, as well as proteins associated with postsynaptic function, respectively **c**-**d**. Volcano plots from total and phospho-proteomics of whole lung homogenates comparing treated versus untreated G2019S KI mice **e**-**f**. Volcano plots key: grey = non-significant, blue = < 0.05 *p*-value, green = > 1.4x log fold change, red = < 0.05 *p*-value and > 1.4x log fold change

Proteomic analysis of brain tissue of 10-week MLi-2-treated mice revealed a number of mitochondrial proteins that showed statistically significant *p*-value (adjusted *p* < 0.05) compared to untreated G2019S KI controls, albeit with modest fold differences between treatment groups (Fig. 7a, b). These include Cytochrome C, NADH:Ubiquinone Oxidoreductase Subunit V3 (Nduvf3), the mitochondrial ATP synthase Atp5g1 and Voltage Dependent Anion Channel 2 (Vdac2) (Fig. 7a, b). Phospho-proteomics analysis revealed a decrease in S58 phosphorylation of the ion transporter Fxyd7 and increase in S109 phosphorylation of the PP2A inhibitor Ensa (Fig. 7b). Gene Ontology analyses revealed enrichment for proteins residing in different mitochondrial compartments as well synaptic proteins (Fig. 7c, d). These data suggest that even though the brain is more resilient in terms of potential endolysosomal defects that may be associated with LRRK2 inhibition, there are small changes in mitochondrial function resulting from chronic LRRK2 inhibition. Volcano plots comparing untreated groups of G2019S KI and wildtype mice showed that a number of proteins had the opposite trend to that seen in the chronic MLi-2 cohort (Fig. S5). For example, the transmembrane ion transporters Sfxn2 and Sfxn3, showed a modest upregulation in kidneys of G2019S LRRK2 compared to wildtype mice and this was rescued in the chronic MLi-2 G2019S LRRK2 cohort (Figs. S5A and Fig. 6a). For some mitochondrial proteins identified in our brain proteomics screen, Cytochrome C, Vdac1, Ndufs3, and Ndufv2 were downregulated in G2019S LRRK2 brain compared to wildtype and this was reversed in the chronic MLi-2 G2019S LRRK2 cohort (Figs. S5B and 7A). This suggests that treatment can significantly alter the expression of mitochondrial proteins in G2019S KI mice in a direction consistent with therapeutic potential.

Finally, 441 total proteins and 189 phosphopeptides were identified in whole lung homogenates as significant with an adjusted *p*-value of < 0.05, of which 14 phosphopeptides and 6 total proteins passed the fold change cutoff of 1.4x in the G2019S MLi-2 treated versus untreated mice (Fig. 7e-f). Of the significant proteins identified, we attempted to validated Myristoylated alanine-rich C-kinase substrate.

(Marcks) due to potential relevance to known LRRK2 pathways. Phosphorylation of Marcks within its ED domain localizes the protein to the plasma membrane of cells, as unphosphorylated protein is released into the cytosol and can bind GTP-bound Rab10 [25], a LRRK2 substrate. Marcks may also be relevant to lung inflammation [26, 27], where LRRK2 has also been implicated [28]. Marcks phosphorylated at S163 was significantly decreased in the MLi-2 treated group and was validated via Western blot (Fig. S6C-D). Additionally, Cyp1a1, a monooxygenase widely distributed via the bloodstream responsible for metabolizing various classes of drugs and carcinogens [29], was upregulated in the lungs of G2019S KI mice treated with MLi-2, which we also validated (Fig. S6C-D). Although these results confirm validity of the lung proteomics results, we did not recover any significant Gene Ontology categories, presumably due to the relative sparsity of significant changes in the lung proteome.

### Validation of endolysosomal, trafficking, and mitochondrial proteins in chronically LRRK2 inhibited mice reveal both a rescue of mutant-driven effects and dysregulatory patterns in vivo

**Fig. 8:**
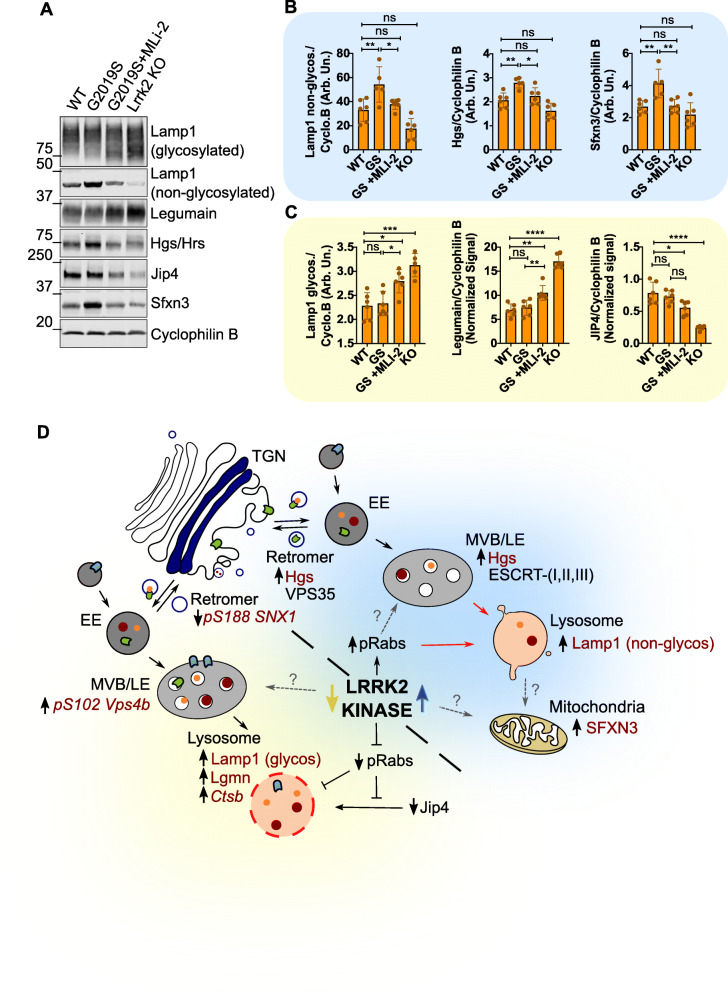
Validation of proteomic hits reveal both rescue of mutant-driven effects and dysregulatory patterns in long-term MLi-2 treated mice. Follow-up validation of proteomics hits in Western blot confirmed differential changes to endolysosomal, trafficking, and mitochondrial proteins. Within these proteins, we observed divergent patterns of therapeutic and dysregulatory effects in the kidneys of 10-week, MLi-2 treated G2019S KI mice **a**. Therapeutic trends were observed in non-glycosylated Lamp1, Hgs, and Sfxn3, in which high expression of these proteins in untreated G2019S KI mice were ameliorated down to wildtype levels with LRRK2 inhibition **b**. We also observed significant increases in glycosylated Lamp1 and Legumain towards levels observed and previously characterized in Lrrk2 KO mice. Additionally, we have shown a significant decrease in Jip4 within the treated mice that were also exacerbated in Lrrk2 KO mice **c**. Based on these discoveries, we have hypothesized divergent mechanisms for LRRK2 hyperactivity and chronic inhibition **d**. The proteins in maroon reflect the proteins identified in our proteomics screens (italicized) and validation on WB (not italicized). The grey dashed arrows depict unknown mechanism/relationship with LRRK2 kinase activity. One-way ANOVA with Tukey’s post hoc; *n* = 6; ****P < 0.0001, **P < 0.005, *P < 0.05; **b** non-glycosylated Lamp1: *F* (3, 19)= 13.58, Hgs: *F* (3, 19)= 13.72, Sfxn3: *F* (3, 19)= 9.323, **c** glycosylated Lamp1: *F* (3, 19)= 13.70, Legumain: *F* (3, 20)= 61.36, Jip4: *F* (3, 20)= 27.39

We next wanted to validate protein hits observed from our proteomics screens in kidney tissue. Through western blot analyses, we found two distinct patterns of effects, one we characterized as beneficial, based on a reverse in protein expression from G2019S KI animal levels back to wildtype levels and the other dysregulatory, based on a mimicking effect comparable to LRRK2 KO animals (Fig. 8a). In the former category, the non-glycosylated form of Lamp1, the ESCRT-0 protein Hgs, and the iron and serine mitochondrial transporter Sfxn3 were all shown to be significantly increased in protein levels in untreated G2019S KI mice compared to wildtype animals. These levels were ameliorated back to wildtype levels after MLi-2 treatment for 10 weeks (Fig. 8b). Conversely, the glycosylated form of Lamp1 and the lysosomal hydrolase Legumain were both significantly increased in the same animals, patterns of which have been previously characterized in LRRK2 KO animals, and is recapitulated here in our LRRK2 KO mice in this cohort [30]. In addition, we also discovered that the motor adaptor protein Jip4 was significantly reduced in LRRK2 KO mice, and this was also seen in G2019S KI mice treated with MLi-2 (Fig. 8c). Jip4 has recently been discovered, by our lab and others, to bind to LRRK2-dependent phosphorylated Rab8a, Rab10, and Rab35 and can be recruited to damaged lysosomal membranes [31, 32]. In the current study, all of these phenotypes are kidney-specific, as no significant difference of these proteins was observed in brain or lung tissues (Fig. S6 A-D). Additionally, these differences were not seen in mice that were treated with MLi-2 for only 10 days (Fig. S6 G-I), suggesting that chronic inhibition of LRRK2 is necessary to see these effects.

## Discussion

In the present study, we have evaluated various markers of LRRK2 inhibition in vivo and demonstrated their utility across brain and peripheral tissues. In both acute and chronic treatment paradigms, we focused on a treatment paradigm that might reasonably mimic clinical applications where the hyperactive mutant of LRRK2, G2019S, expressed at the endogenous level is chronically depressed to that of wildtype animals. We performed proteomics to examine the molecular effects of chronic LRRK2 inhibition and identified changes to the endolysosomal, trafficking, and mitochondrial systems in kidneys at the total protein level as well as phosphorylation events. In addition, we noted a modest but statistically significant change in mitochondrial proteins, most notably enriched within the mitochondrial electron transport chain, in the brains of MLi-2 treated G2019S mice.

In our studies in mouse tissue, measuring LRRK2 phosphorylation, particularly the pS1292 autophosphorylation site, provides the most reliable marker of both G2019S-induced hyperactivity and MLi-2 dependent inhibition. Whether this can be extrapolated to human clinical samples is unclear as current tools have not yet allowed for robust detection of pS1292 LRRK2 in peripheral blood, although some studies have demonstrated its efficacy in urinary and CSF exosomes [33, 34]. Using the commercially available pRab antibodies, we found that S106 Rab12 phosphorylation pattern resembled that of pS1292 LRRK2 most closely across experiments, in that G2019S KI mice displayed hyperphosphorylation that was ameliorated with MLi-2 treatment. This is in contrast to the hyperphosphorylation pattern of other Rab substrates, which have been demonstrated in mutations within the GTPase domain but not for G2019S [35, 36]. Thus, for G2019S-carriers and potentially other PD cases, detection of pRab12 has potential as a biomarker for LRRK2 activity and inhibition.

With regard to pRab10 and pRab29, we show that their levels do not correlate with MLi-2 treatment in vivo, particularly within the chronic in-diet treatment paradigm. We hypothesize a few reasons for this. Firstly, Vieweg et al. reported a PINK1-dependent S111 phospho-site conserved across multiple Rab substrates that can negatively regulate subsequent LRRK2-dependent phosphorylation by preventing Switch-II binding with its GEF, as demonstrated with pT72 Rab8a in vitro [37]. It is possible that Rab10 and Rab29 LRRK2-dependent phosphorylation could be regulated through a similar unknown mechanism and thus no change to basal levels of phosphorylation are observed with LRRK2 inhibition. Additionally, other unknown kinases that phosphorylate these Rabs on the same residue as LRRK2 and normally compete with LRRK2 for binding may be able to phosphorylate these proteins more readily under conditions of LRRK2 inhibition. Lastly, we recently reported differing localization patterns between pS106 Rab12 and pT73 Rab10 when artificially trapping LRRK2 to different cellular membranes. Independent of which membrane LRRK2 was directed to intracellularly, pRab12 was found to colocalize with LRRK2, whereas pRab10 was primarily detected at perinuclear lysosomes and Golgi after directing LRRK2 to these membranes [38]. This falls in line with the idea that a more complex mechanism surrounds the Rab10:LRRK2 interaction and thus any influence LRRK2 has on Rab10 is more conditional compared to Rab12. These hypotheses could explain the lack of dephosphorylation of T73 Rab10 in the presence of MLi-2, and further exploration of other kinases and phosphatases that regulate Rab GTPase phosphorylation, such as PPM1H [39], will be necessary to elucidate the functional dynamics between Rab10 and LRRK2 across different organelles.

Using these two validated biomarkers, pS1292 LRRK2 and pS106 Rab12, a 60 mg/kg/day dosing of MLi-2 in G2019S KI mice via in-diet dosing for 10 days or 10 weeks was shown to be sufficient to revert the hyperactive mutation approximately to wild type levels. Such an approach could be helpful in identifying similar dosing regimens in humans. Importantly, we show that measurements in some peripheral tissues, particularly kidney, reflect events in the brain, the target organ for any PD clinical trial. However, we observed that after long-term chronic MLi-2 in-diet dosing, S1292 LRRK2 and S106 Rab12 phosphorylation was significantly lower than wildtype levels in lung. It is possible that lung tissue is more sensitive to LRRK2 inhibition than other tissues considered here and has also been suggested in a recent study investigating the toxicological and morphological effects of LRRK2 inhibition on nonhuman primate lungs, in which high doses of three structurally distinct LRRK2-specific inhibitors, including MLi-2, induced vacuolated cytoplasm in type II pneumocytes, however no pulmonary deficits were observed and the phenotype was quickly reversed after cessation of treatment [16]. These data were recently recapitulated in mice by Bryce and colleagues. In this study the authors treated wildtype mice with in-diet MLi-2 for six months and showed that the levels of prosurfactant protein C were increased early on in a 60 mg/kg/day dosing regimen and decreased back to control levels after 90 days [40]. These reports suggest that there is a lung-specific LRRK2-dependent mechanism that is affected by chronic LRRK2 inhibition. Based on these data, we probed our 10-day and 10-week treatment cohorts for proSP-C in lung tissue via Western blot. We found no changes at either timepoints in G2019S KI mice treated with MLi-2, although a slight but significant elevation of proSP-C was observed in the Lrrk2 KO animals (Fig. S6C-F). This would imply that the absence of LRRK2 has an effect of proSP-C levels and MLi-2 treatment would have a more dramatic effect on wildtype mouse lung tissue than in a G2019S KI. A 60 mg/kg-dosing inhibition on wildtype activity could potentially mimic LRRK2 deficiency and this is not recapitulated in our regimen where we aimed to lower G2019S LRRK2 activity to wildtype levels. Another important aspect to note is the potential limitation in detecting surfactant changes by western blot, due to their ~ 90% lipid/10% composition and further lipid analysis would be more informative in comparing wildtype and G2019S hyperactive kinase inhibition for possible differences. Overall, these data suggest that while LRRK2 inhibitors can be used to diminish hyperactive kinase to wild type levels of activity, there are tissue-specific differences in activity and stability of the protein that may be important to consider when using peripheral engagement to infer brain inhibition.

Unbiased proteomic screens revealed significant differences in endolysosomal, trafficking, and mitochondrial proteins in the kidneys of treated compared to untreated G2019S KI animals. We identified two subsets of proteins, those that suggest a therapeutic effect and those that are dysregulatory, with long-term chronic LRRK2 inhibition in vivo. Strikingly, all differential changes were specific to our 10-week treatment cohort compared to our 10-day cohort. This suggests that these molecular changes are specific to the length of LRRK2 inhibition. Thus, future preclinical efforts are needed that include long-term inhibitor treatment in order to understand what may happen in human disease. Proteins altered towards a therapeutic effect included non-glycosylated Lamp1, Hgs, and Sfxn3, whereas glycosylated Lamp1, leguman, and Jip4 showed likeness to LRRK2 KO expression levels. Based off of these proteins, we hypothesize a mechanism for the endolysosomal trafficking pathway in the context of both LRRK2 kinase hyperactivity and chronic inhibition, in which both dysfunctional LRRK2 kinase states converge on the same pathway through different means depicted in Fig. 8d.

In recent years, the role of LRRK2 at the lysosome has been a focus of research that explores how mutations in this gene cause disease. Multiple labs have now reported lysosomal dysfunction in both LRRK2 KO and G2019S mutant models, LRRK2 recruitment to lysosomes under stress as well as alpha-synuclein accumulation in Lrrk2 deficient models [10, 17, 32, 41]. We show here that increased non-glycosylated Lamp1 found in G2019S KI mice was ameliorated back to wildtype levels after 10-week MLi-2 treatment. Glycosylation of Lamp1 is an important feature in maintaining lysosomal stability and protein transport across its membrane [42]. Glycosylation is initiated in the rough endoplasmic reticulum and moves through the Golgi before reaching lysosomes. Thus, an increase in the non-glycosylated form suggests either that there has been an incomplete maturation of Lamp1 before reaching the lysosomes or an upregulation in Lamp1 translation due to increased lysophagy in the kidneys of G2019S KI mice. Premature lysophagy and redirection of proteins for degradation to the remaining active lysosomes may then overwhelm the system and lead to lysosomal stress. In contrast, an increase in glycosylated Lamp1 has been previously characterized in Lrrk2 KO mice and in the postmortem brains of sporadic PD patients [30, 43] and is shown here in G2019S KI mice treated with MLi-2 and LRRK2 KO mice. This suggests that there can be lysosomal buildup and protein accumulation in the absence of active LRRK2. Both alterations of Lamp1 suggest LRRK2 kinase activity plays a role in the integrity of lysosomes, and chronic inhibition of LRRK2 may restore healthy levels of the non-glycosylated form, while perpetuating a shift that mimics a loss of function effect in its glycosylated form. This suggests that treatment with LRRK2 kinase inhibitors may require careful consideration of both premature lysophagy and lysosomal protein accumulation.

In brain tissue, chronic MLi-2 treatment in G2019S KI mice showed a modest but significant upregulation of mitochondrial proteins compared to their untreated counterparts (Fig. 7a). Production of reactive oxygen species (ROS), mitochondrial elongation, decreased ATP production and mitochondrial DNA damage have been reported in G2019S PD patient-derived skin biopsies and G2019S-LRRK2 in vitro models [44–48]. In brain tissue, the majority of mitochondrial proteins found in our screens were associated with the respiratory chain Complex I and III. Further studies are needed in order to elucidate the effects of LRRK2 on the respiratory chain system. However, these results do not suggest a potential concern for LRRK2 kinase inhibition affecting mitochondrial function as in general they respond in the opposite direction of effect to the pathogenic G2019S mutation, i.e. are in the therapeutic direction. A limitation of this study is that whole brain homogenate was used for the proteomics analysis which may have limited our ability to determine regional- and cell type-dependent LRRK2 mechanisms. Further studies focused on regional and cell-specific parameters can shed a deeper mechanistic insight into the molecular consequences of LRRK2 inhibition in the brain.

## Conclusion

The aim of the present study was twofold: to assess the suitability of new antibodies of known LRRK2 substrates as biomarkers for LRRK2 kinase inhibition and to evaluate what molecular effects are observed when LRRK2 is chronically inhibited. We found that pRab12 is a reliable readout of LRRK2 activity and inhibition, and that phosphorylation patterns seen in the periphery reflect events in the brain. Additionally, our study highlights the molecular effects of chronic inhibition that remain to be explored and warrant further investigation in the light of the ongoing clinical trials. What remains to be determined is which of these effects are truly protective or damaging, and how relevant they are to what happens in human disease.

As of yet, there continues to be a significant unmet medical need in the field of neurodegeneration for effective, long lasting treatments that either halt or slow disease progression. Existing drug treatments for PD target the symptoms of the disease rather than cause, and do not modify disease course. Clinical trials are currently underway to assess the safety and efficacy of LRRK2-specific kinase inhibition in PD patients [49]. In parallel with these trials, future work will need to elucidate the underlying disease mechanisms that cause PD and how LRRK2 mutations ultimately lead to neurodegeneration. Understanding the pathobiological role of LRRK2 kinase activity will greatly aid in the development and optimization of therapeutic strategies.

## Supplementary Information

**Additional file 1 Table S1:** Table of antibody summary. All antibodies used in this study are listed with catalog numbers, and working concentrations used. IgG concentrations are supplied in brackets.

**Additional file 2 S2**. Characterization of pT73 Rab10 [MJF-R21 and MJF-R21–22-5] antibodies. (A, B) HEK293FT cells transiently expressing LRRK2 mutant constructs were analyzed for endogenous Rab10 phosphorylation [MJF-R21]. The LRRK2 genetic variants R1441G, Y1699C and I2020T invoked the highest increase in Rab10 phosphorylation compared to WT LRRK2, while the kinase-dead K1906M LRRK2 construct conferred a significantly lower pRab10 levels compared to WT. (C, D) HEK293FT cells transiently expressing LRRK2 genetic variants following Rab10 siRNA knock-down were probed for Rab10. Rab10 levels revealed successful knock-down of endogenous Rab10 compared to non-targeting controls. The pT73 Rab10 [MJF-R21] antibody detected a band at ~ 24 kDa, which was not detected in the Rab10 siRNA groups, suggesting specificity for Rab10. (E, F) In a similar experiment to (C), Rab10 expression was knocked-down in primary astrocytes from WT and homozygous G2019S LRRK2 knock-in mice. Rab10 knock-down was followed by a significant decrease in pT73 Rab10 signal using the MJF-R21 antibody (E, F). (G, H) Primary astrocytes treated with 1 μM of MLi-2 for 90 min showed that both pT73 Rab10 antibodies (MJF-R21 and MJF-R21–22-5) show significantly decreased levels of phosphorylation compared to control cells. Quantitation of phosphorylation levels in D and F are presented as raw pT73 intensity normalized to loading while B and H represent T73 phosphorylation signal over total Rab10 levels (B: one-way ANOVA with Tukey’s post hoc, ****P < 0.0001, ***P < 0.0002, *n* = 3, *F* (8, 18)= 45.45. D: two-way ANOVA with Sidak’s multiple comparisons test; LRRK2 construct, *p* < 0.0001, *F* (2, 12)=30.73; siRNA, *p* < 0.0001, *F* (1, 16)=550.0, *n* = 3. F: two-way ANOVA with Sidak’s multiple comparisons test; LRRK2 construct, *p* = 0.7361, *F* (1, 8)=0.1218; siRNA, *p* < 0.0001, *F* (1, 8)=56.29, *n* = 3. H: one-way ANOVA with Tukey’s post hoc, ****P < 0.0001, *n* = 3; *F* (3, 15)= 160.3). **S3**. Characterization of pS106 Rab12 [MJF-25-9] and pT71 Rab29 [MJF-R24–17-1] antibodies. (A) HEK293FT cells transiently expressing LRRK2 genetic variants following siRNA knock-down of Rab12 were analyzed for Rab12 phosphorylation. The pS106 Rab12 antibody showed a strong band at ~ 25 kDa with LRRK2 transfection, which was significantly decreased by Rab12 siRNA compared to non-targeting control (A, B). (C, D) HEK293FT cells transiently expressing LRRK2 mutant constructs were analyzed for endogenous pT71 Rab29. Endogenous Rab29 was found hyperphosphorylated in R1441C, R1441G, Y1699C, G2019S and I2020T LRRK2 expressing cells, compared to WT LRRK2. (E, F) siRNA Rab29 knockdown resulted in a significant decrease in pT71 Rab29 signal in HEK293FT cells over-expressing LRRK2 mutants compared to non-targeting controls. (G) The T71 Rab29 antibody tested did not detect a band in cells expressing the T71A Rab29 phospho-null variant while a strong band was observed in cells expressing WT Rab29 construct, in co-expression with LRRK2 constructs. (H) Lung tissue collected from Rab29 KO mice tissue showed no pT71 Rab29 signal, whereas G2019S Lrrk2 knock-in mice showed increase in Rab29 phosphorylation, compared to WT mice. Quantitation of phosphorylation levels is presented as S106 normalized to loading (cyclophilin B levels; in B), T71 over total Rab29 (in D) or normalized to loading (F). (B: two-way ANOVA with Sidak’s multiple comparisons test; LRRK2 construct, *p* = 0.0083, *F* (2, 12)=7.329; siRNA, *p* < 0.0001, *F* (1, 16)=367.8, *n* = 3. D: one-way ANOVA with Tukey’s post hoc, ****P < 0.0001, ***P < 0.0002, ** < 0.004, *F* (8, 18)= 528.0, *n* = 3. F: two-way ANOVA with Sidak’s multiple comparisons test; LRRK2 construct, *p* < 0.0001 *F* (2, 12)=31.83; siRNA, *p* < 0.0001, *F* (1, 16)=191.1, *n* = 3. Mean values with SD bars shown). **S4**. pT73 Rab10 and pT71 Rab29 phosphorylation following chronic MLi-2 treatment. Full blots from the 10-day and 10-week chronic studies are shown to account for animal-to-animal variability (A, B). pT73 Rab10 across tissues and treatment groups show large variability with no discernable trend after LRRK2 inhibition (C, E). Rab29 T71 phosphorylation levels do not respond to treatment across tissues and time points, with exception to brain tissue of the short-term cohort (D, F). (G) Pearson’s correlation coefficients were conducted to test confounding variables within the acute and chronic paradigms of this study with regard to pT73 Rab10 variability, of which relationships with dose of MLi-2, length of experiment, age of mice, gender, and batch of tissue processing were not found. One-way ANOVA with Tukey’s post hoc; *n* = 6; ****P < 0.0001, ***P < 0.0002, **P < 0.005, *P < 0.05; (C) F (11, 60) = 28.56, (D) F (11, 60) = 43.20, (E) F (11, 59) = 24.70, (F) F (11, 60) = 8.279. **S5**. Proteomic analysis of changes in brain, kidney, and lung between untreated G2019S LRRK2 and wildtype mouse cohorts. Volcano plots depicting differences in levels of total proteins in kidney (A), brain (B), and lung (C) between untreated G2019S KI and wildtype mice. A number of proteins show subtle changes involved in iron transport and mitochondrial homeostasis are highlighted. Lung hits were not preferentially enriched for any particular cellular component. Volcano plots key: grey = non-significant, blue = < 0.05 *p*-value, green = > 1.4x log fold change, red = < 0.05 p-value and > 1.4x log fold change. **S6**. Kidney hits are tissue-specific with proteins identified in the 10-week cohort and do not show differential change in the 10-day cohort. Proteins identified in the kidney proteomics and validated via Western blot were also investigated in the brain and lung tissues of the 10-week cohort animals. We found that these proteins (Jip4, Lamp1, HGS/Vps27, Sfxn3 and legumain) were not statistically altered in the G2019S KI animals, neither MLi-2 treated nor untreated, in brain and lung (A-D). This implies a kidney-specific phenotype of these altered lysosomal and other membrane proteins. Additionally, we explored two of the mitochondrial proteins found in the brain total proteomics, Atp5mc1 and Cytochrome C. No difference was observed in G2019S MLi-2 treated mice compared to any other group (C-D). Additionally, proteins Cyp1a1 and pS163 Marcks identified in the lung proteomics were validated via Western blot analysis (C-D). Prosurfactant C was measured in both 10-week (C) and 10-day (E) cohorts in lung tissue of which Lrrk2 KO animals showed a significant elevation while G2019S KI animals receiving MLi-2 treatment were not affected in either cohort (C-F). Proteins identified as statistically significant in the 10-week cohort were also investigated in the 10-day cohort in order to determine if the effects observed occur after continued use of LRRK2 inhibitor long-term as opposed to more immediate effects (after 10 days). Western blot analysis confirmed that both therapeutic and dysregulatory effects were not observed in the 10-day cohort of treated animals (G-I). This suggests that the endolysosomal, trafficking, and mitochondrial changes we observed in our proteomics screen are effects of long-term treatment with MLi-2 in G2019S KI mice. Interestingly, the increase in protein expression in our untreated G2019S KI that was observed in our 10-week cohort, specifically non-glycosylated Lamp1, Hgs, and Sfxn3 was not seen in our 10-day cohort (H). Our current hypothesis is that these changes in protein expression are age-dependent in the context of the G2019S mutation in the mouse model, as the 10-week animals are double the age of the 10-day cohort at time of sacrifice. One-way ANOVA with Tukey’s post hoc; *n* = 6; ****P < 0.0001, ***P < 0.0002, **P < 0.005, *P < 0.05; (B) Jip4: F (3, 20)= 0.8688, glycosylated Lamp1: F (3, 20)= 0.4520, HGS: F (3, 20)= 0.1410, Legumain: F (3, 20)= 0.2312, Atp5mc1: F (3, 20)= 1.953, Sfxn3: F (3, 20)= 0.2285, Cytochrome C: F (3, 20)= 1.698, (D) F (3, 20)= Jip4: F (3, 20)= 1.772, HGS: F (3, 20)= 0.1636, Legumain: F (3, 20)= 0.1724, Sfxn3: F (3, 20)= 1.156, pS163 Marcks: F (3, 20)= 4.739, Cyp1a1: F (3, 20)= 208.2, ProSP-C: F (3, 20)= 4.495, (F) ProSP-C: F (3, 20)= 4.185, (H) non-glycosylated Lamp1: F (3, 20)= 2.802, Hgs: F (3, 20)= 5.173, Sfxn3: F (3, 20)= 3.002, (I) glycosylated Lamp1: 4.702, Legumain: F (3, 20)= 4.409, Jip4: F (3, 20)= 12.35. **S7**. LRRK2 protein interactome from long-term chronic kidney proteomics. The 149 mouse kidney protein hits from chronically treated G2019S KI mice were converted to their human orthologues for LRRK2 interactome analyses using PINOT online tool. Seventy-six matches were identified within the LRRK2 interactome. Five of the matching proteins (AHCYL1, EEF2, HSP90AA1, HSP90AB1 and RANBP2) were present in the first layer while 71 in the second layer of LRRK2 interactions (one step from direct LRRK2 interactors). Two visualizations are shown: (A) highlights the proteins from total or phospho-proteomics runs, (B) depicts primary and secondary interactome layers.

**Additional file 3:.**

**Additional file 4:.**

**Additional file 5:.**

## Data Availability

Raw proteomics data can be obtained from the corresponding author.

## Abbreviations

Ci-M6PR: Cation-independent mannose 6 phosphate receptor
Ctsb: Cathepsin B
Glb1: β-galactosidase
Glut4: Glucose transporter 4
Gns: N-acetylglucosamine-6-sulfatase
Hgs: Hepatocyte growth factor-regulated tyrosine kinase substrate
Jip4: JNK-interacting protein 4
Lamp1: Lysosomal-associated membrane protein 1
Lgmn: Legumain (or AEP, asparaginyl endopeptidase)
LRRK2: Leucine-rich repeat kinase 2
MLi-2: Merch LRRK2 inhibitor 2
Ndufa2: NADH dehydrogenase [ubiquinone] 1 alpha subcomplex subunit 2
Ndufs3: NADH dehydrogenase [ubiquinone] iron-sulfur protein 3
Ndufv3: NADH dehydrogenase [ubiquinone] flavoprotein 3
PD: Parkinson’s disease
PINK1: PTEN-induced kinase-1
PPM1H: Protein phosphatase 1H
ROS: Reactive oxygen species
Sfxn3: Sideroflexin 3
Snx: Sorting nexin (1, 5, 9, 12, 17)
TGN: *Trans*-Golgi network
Uqcrc1: Cytochrome b-c1 complex subunit 1
Uqcrc2: Cytochrome b-c1 complex subunit 2
Vdac2: Voltage Dependent Anion Channel 2
Vps4b: vacuolar sorting protein 4b
