# Supplementary material for: Preclinical modeling of chronic inhibition of the Parkinson’s disease associated kinase LRRK2 reveals altered function of the endolysosomal system in vivo
Source: Mol Neurodegener. 2021 Mar 19;16:17. doi: 10.1186/s13024-021-00441-8 (PMC7977595; doi:10.1186/s13024-021-00441-8)
Supplement: Supplementary file 1 — Additional file 1 Table S1: Table of antibody summary. All antibodies used in this study are listed with catalog numbers, and working concentrations used. IgG concentrations are supplied in brackets. [file 13024_2021_441_MOESM1_ESM.docx]

**S1. Table of antibody summary.**

| Antibody | Species | Catalog # | Working dilution |
| --- | --- | --- | --- |
| pS1292 LRRK2 [MJF-19-7-8] | Rabbit; monoclonal | Ab203181 (Abcam) | 1:2000  [1.15mg/ml] |
| pS935 LRRK2  [UDD2 10(12)] | Rabbit; monoclonal | Ab133450 (Abcam) | 1:2000  [1.019mg/ml] |
| LRRK2 [MJFF2] | Rabbit; monoclonal | Ab133474 (Abcam) | 1:2000  [0.66mg/ml] |
| Cyclophilin B | Rabbit; polyclonal | Ab16045 (Abcam) | 1:2000  [1mg/ml] |
| pT73 Rab10 [MJF-R21] | Rabbit; monoclonal | Ab230261 (Abcam) | 1:2000  [0.54mg/ml] |
| pT73 Rab10 [MJF-R21-22-5] | Rabbit; monoclonal | Ab241060 (Abcam) | 1:2000  [0.56mg/ml] |
| Rab10 [MJF-R23] | Rabbit; monoclonal | Ab237703 (Abcam) | 1:2000  [0.56mg/ml] |
| pS106 Rab12 [MJF-R25-9] | Rabbit; monoclonal | Ab256487 (Abcam) | 1:2000  [0.62mg/ml] |
| Rab12 | Rabbit; polyclonal | 18843-1-AP (Proteintech) | 1:1000  [51µg/150µl] |
| pT71 Rab29 [MJF-R24-17-1] | Rabbit; monoclonal | Ab241062 (Abcam) | 1:1000  [0.54mg/ml] |
| Rab29 [MJF-R30-104] | Rabbit; monoclonal | Ab256527 (Abcam) | 0.5μg/ml  [1.07mg/ml] |
| Rab29 [MJF-R30-124] | Rabbit; monoclonal | Ab256526 (Abcam) | 0.5μg/ml  [0.68mg/ml] |
| Legumain | Rabbit; monoclonal | 93627S (Cell Signaling) | 1:1000 |
| Jip4 | Rabbit; monoclonal | 5519S (Cell Signaling) | 1:1000  [45µg/150µl] |
| Hgs | Rabbit; polyclonal | 10390-1-AP (Proteintech) | 1:1000  [45µg/150µl] |
| Sfxn3 | Rabbit; polyclonal | 15156-1-AP (ThermoFisher) | 1:1000  [105µg/150µl] |
| Lamp1 | Rabbit; monoclonal | 3243S (Cell Signaling) | 1:1000 |
| Atp5mc1 | Mouse; monoclonal | ab119686 | 1:1000  [1mg/ml] |
| Cytochrome C | Mouse; monoclonal | ab13575 | 1:1000  [1mg/ml] |
| pS163 MARCKS | Rabbit; monoclonal | 11992S (Cell Signaling | 1:1000 |
| Cyp1a1 | Rabbit; polyclonal | 13241-1-AP (ThermoFisher) | 1:1000  [500µg/ml] |
| ProSP-C | Rabbit; polyclonal | Ab90716 (Abcam) | 1:1000  [0.9mg/ml] |
