# Supplementary figures and images for: Preclinical modeling of chronic inhibition of the Parkinson’s disease associated kinase LRRK2 reveals altered function of the endolysosomal system in vivo
Source: Mol Neurodegener. 2021 Mar 19;16:17. doi: 10.1186/s13024-021-00441-8 (PMC7977595; doi:10.1186/s13024-021-00441-8)

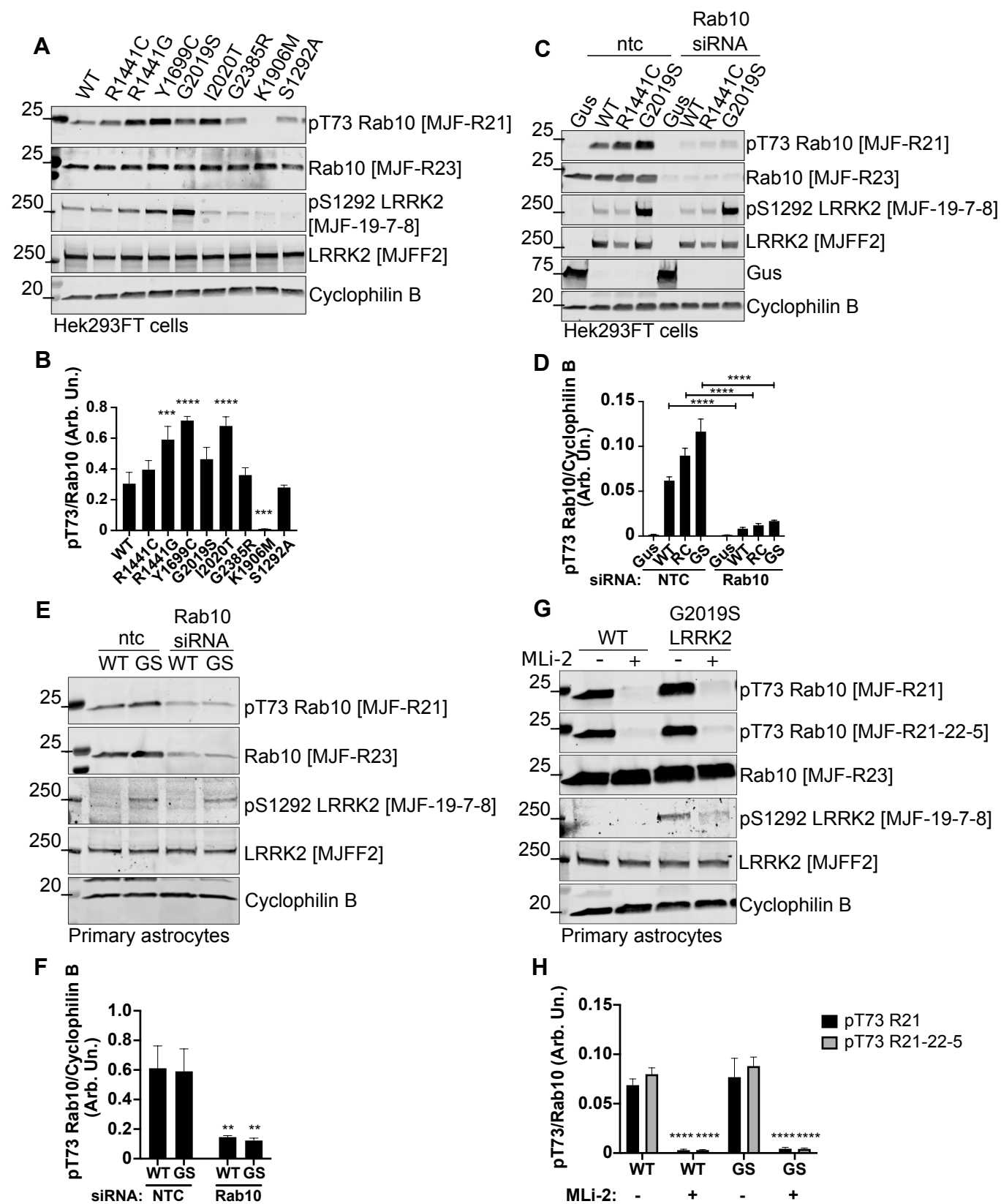

Figure S2

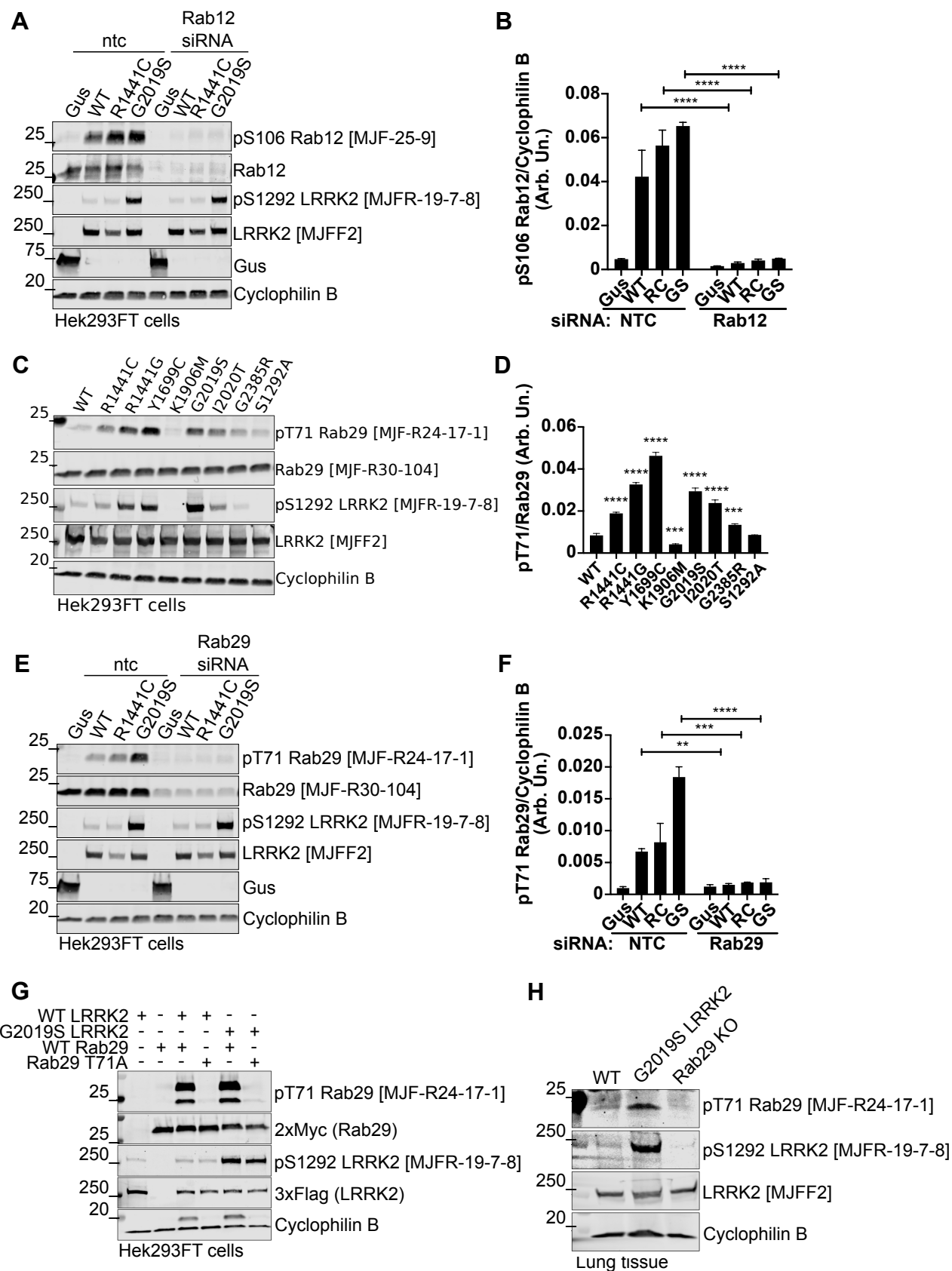

Figure S3

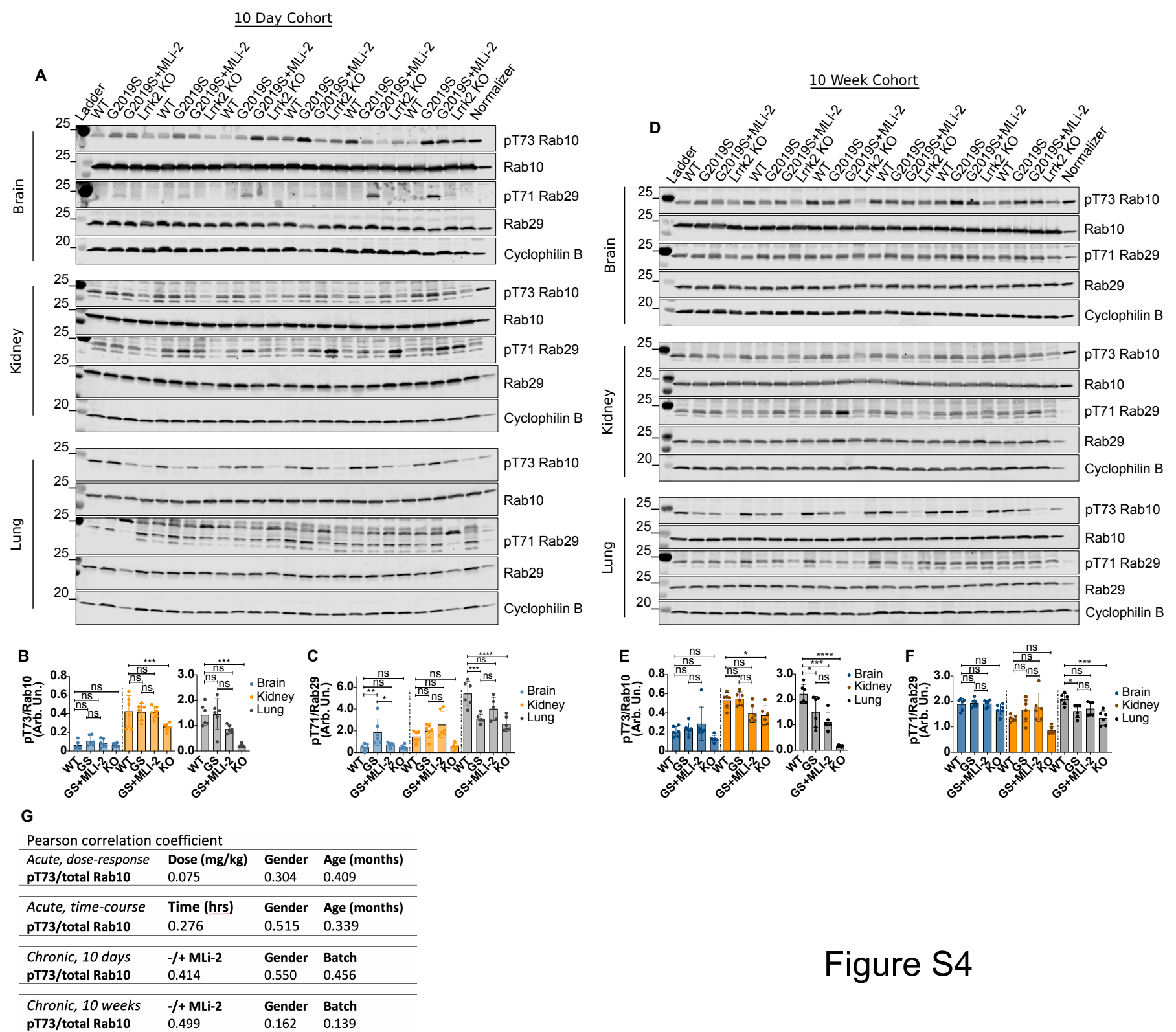

Figure S4

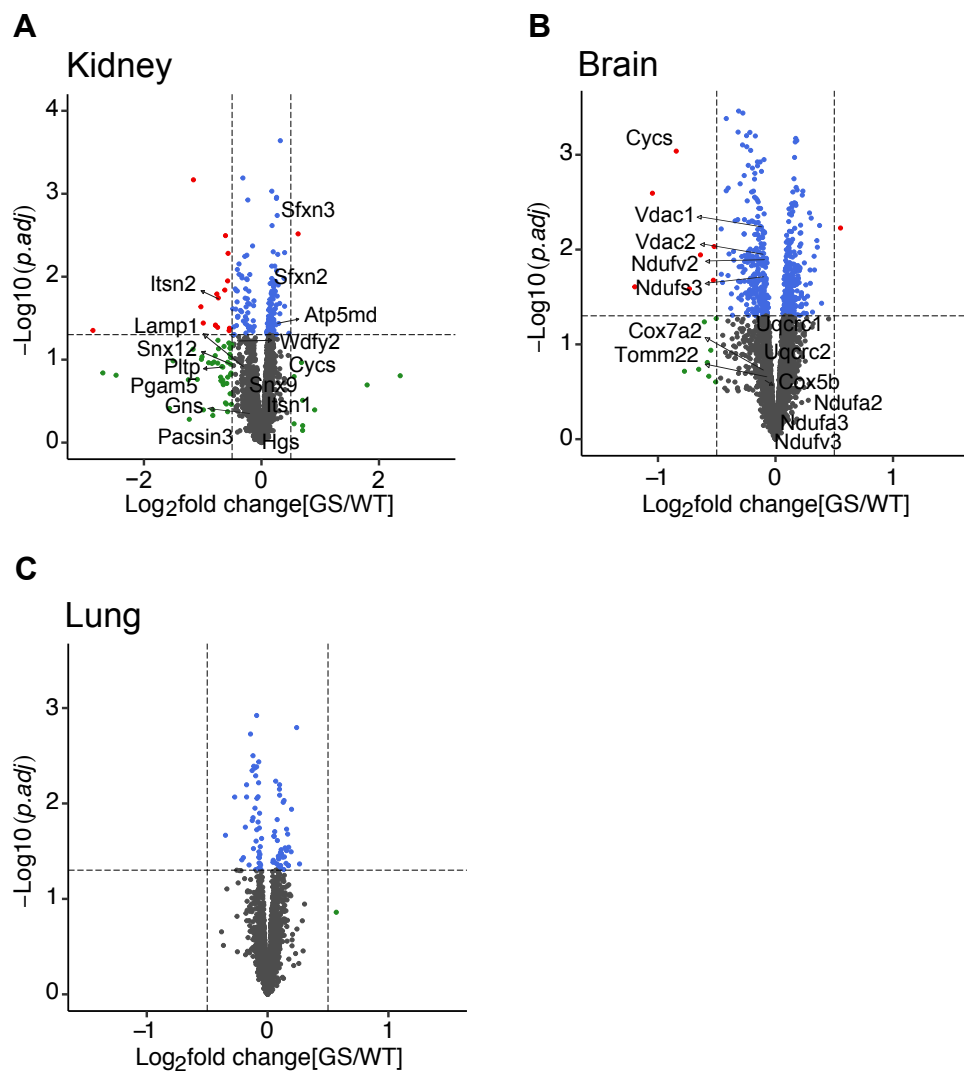

Figure S5

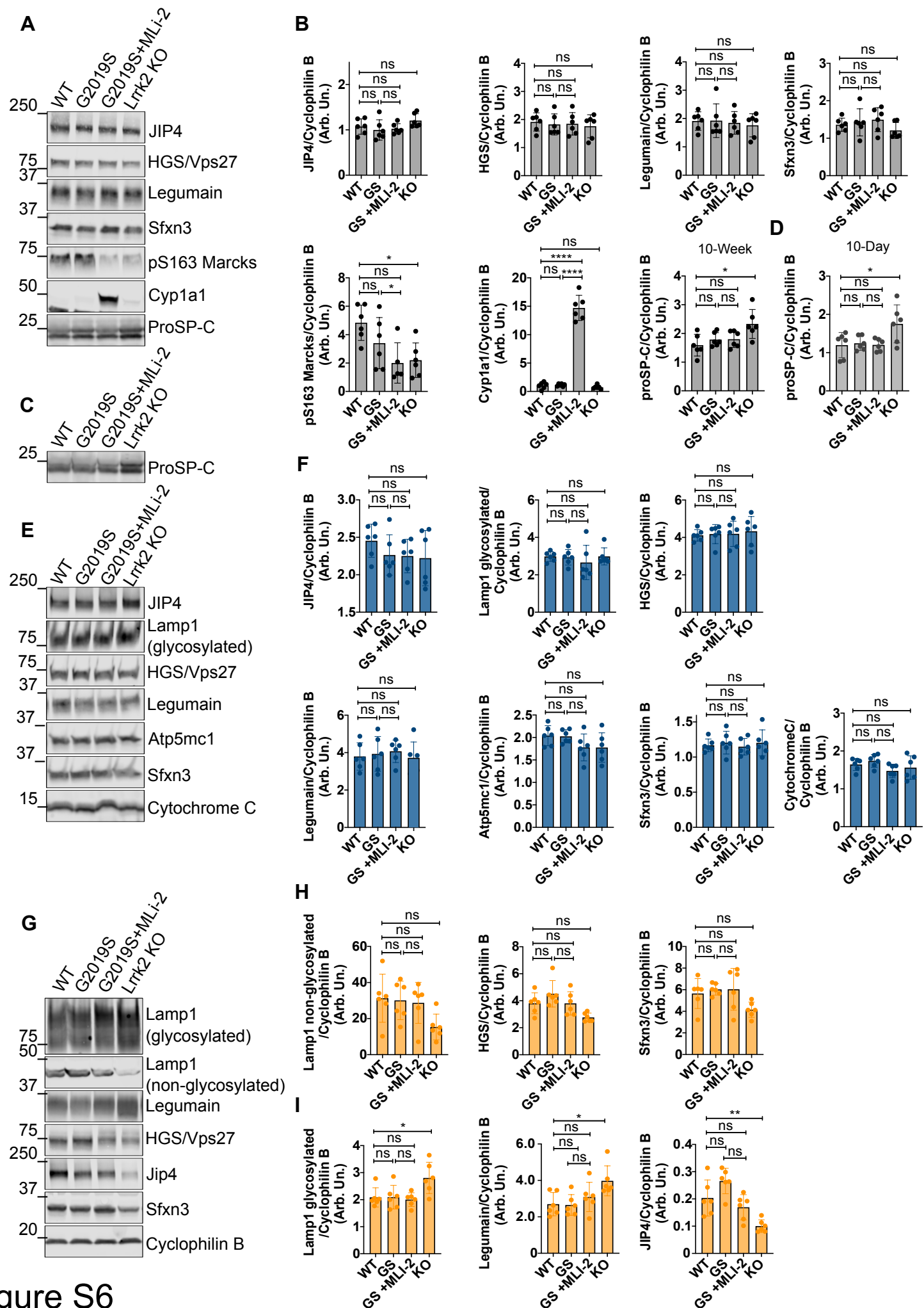

Figure S6

A

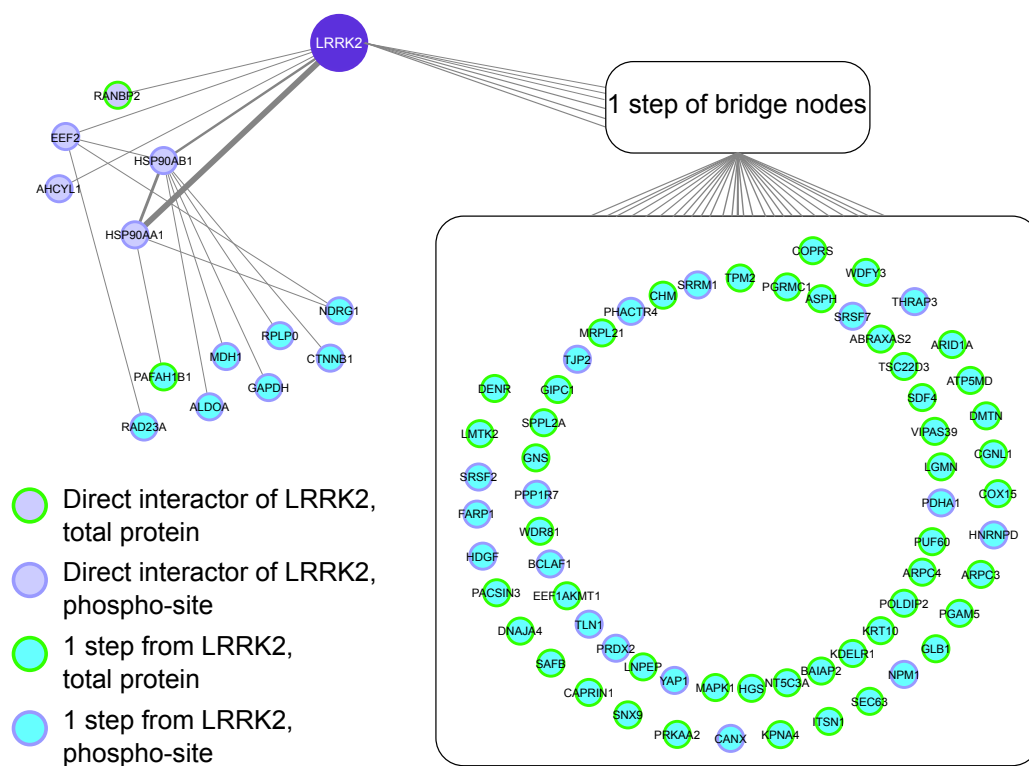

B

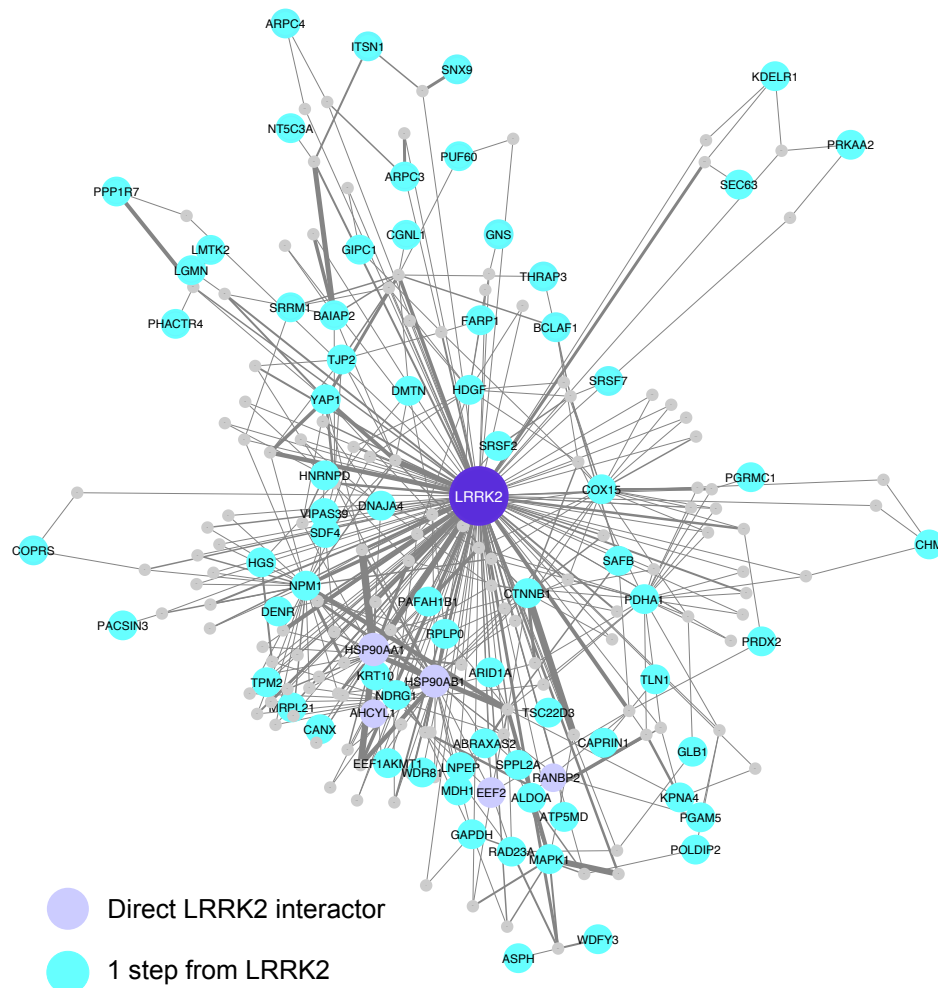

Figure S7

Supplement: Supplementary file 2 — Additional file 2 S2. Characterization of pT73 Rab10 [MJF-R21 and MJF-R21–22-5] antibodies. (A, B) HEK293FT cells transiently expressing LRRK2 mutant constructs were analyzed for endogenous Rab10 phosphorylation [MJF-R21]. The LRRK2 genetic variants R1441G, Y1699C and I2020T invoked the highest increase in Rab10 phosphorylation compared to WT LRRK2, while the kinase-dead K1906M LRRK2 construct conferred a significantly lower pRab10 levels compared to WT. (C, D) HEK293FT cells transiently expressing LRRK2 genetic variants following Rab10 siRNA knock-down were probed for Rab10. Rab10 levels revealed successful knock-down of endogenous Rab10 compared to non-targeting controls. The pT73 Rab10 [MJF-R21] antibody detected a band at ~ 24 kDa, which was not detected in the Rab10 siRNA groups, suggesting specificity for Rab10. (E, F) In a similar experiment to (C), Rab10 expression was knocked-down in primary astrocytes from WT and homozygous G2019S LRRK2 knock-in mice. Rab10 knock-down was followed by a significant decrease in pT73 Rab10 signal using the MJF-R21 antibody (E, F). (G, H) Primary astrocytes treated with 1 μM of MLi-2 for 90 min showed that both pT73 Rab10 antibodies (MJF-R21 and MJF-R21–22-5) show significantly decreased levels of phosphorylation compared to control cells. Quantitation of phosphorylation levels in D and F are presented as raw pT73 intensity normalized to loading while B and H represent T73 phosphorylation signal over total Rab10 levels (B: one-way ANOVA with Tukey’s post hoc, ****P < 0.0001, ***P < 0.0002, n = 3, F (8, 18)= 45.45. D: two-way ANOVA with Sidak’s multiple comparisons test; LRRK2 construct, p < 0.0001, F (2, 12)=30.73; siRNA, p < 0.0001, F (1, 16)=550.0, n = 3. F: two-way ANOVA with Sidak’s multiple comparisons test; LRRK2 construct, p = 0.7361, F (1, 8)=0.1218; siRNA, p < 0.0001, F (1, 8)=56.29, n = 3. H: one-way ANOVA with Tukey’s post hoc, ****P < 0.0001, n = 3; F (3, 15)= 160.3). S3. Characterization of pS106 Rab12 [MJF-25-9] [file 13024_2021_441_MOESM2_ESM.pdf]
