# Supplementary material for: Preclinical modeling of chronic inhibition of the Parkinson’s disease associated kinase LRRK2 reveals altered function of the endolysosomal system in vivo
Source: Mol Neurodegener. 2021 Mar 19;16:17. doi: 10.1186/s13024-021-00441-8 (PMC7977595; doi:10.1186/s13024-021-00441-8)
Supplement: Supplementary file 3 — Additional file 3:. [file 13024_2021_441_MOESM3_ESM.docx]

**Hemi-brain** labeling layout:

| **TMT-Run1** |  | **TMT-Run2** |  | **TMT-Run3** |  | **TMT-Run4** |  |
| --- | --- | --- | --- | --- | --- | --- | --- |
| 126 | Pool-1 | 126 | Pool-2 | 126 | Pool-3 | 126 | Pool-4 |
| ~~127N~~ |  | 127N | 10W-WT2 | 127N | 10D KO5 | 127N | 10W KO6 |
| ~~127C~~ |  | 127C | 10W GS2 | 127C | 10D GS+5 | 127C | 10W GS+6 |
| ~~128N~~ |  | 128N | 10W GS+2 | 128N | 10D GS5 | 128N | 10W GS6 |
| 128C | 10D-WT1 | ~~128C~~ |  | 128C | 10D-WT5 | 128C | 10W-WT6 |
| 129N | 10D GS1 | ~~129N~~ |  | 129N | 10W KO4 | 129N | 10D KO6 |
| 129C | 10D GS+1 | ~~129C~~ |  | 129C | 10W GS+4 | 129C | 10D GS+6 |
| 130N | 10D KO1 | 130N | 10W KO2 | ~~130N~~ |  | 130N | 10D GS6 |
| 130C | 10W-WT1 | 130C | 10D-WT3 | ~~130C~~ |  | 130C | 10D-WT6 |
| 131N | 10W GS1 | 131N | 10D GS3 | ~~131N~~ |  | 131N | 10W-KO5 |
| 131C | 10W GS+1 | 131C | 10D GS+3 | 131C | 10W GS4 | ~~131C~~ |  |
| 132N | 10W KO1 | 132N | 10D KO3 | 132N | 10W-WT4 | ~~132N~~ |  |
| 132C | 10D-WT2 | 132C | 10W-WT3 | 132C | 10D KO4 | ~~132C~~ |  |
| 133N | 10D GS2 | 133N | 10W GS3 | 133N | 10D GS+4 | 133N | 10W GS+5 |
| 133C | 10D GS+2 | 133C | 10W GS+3 | 133C | 10D GS4 | 133C | 10W GS5 |
| 134N | 10D KO2 | 134N | 10W KO3 | 134N | 10D-WT4 | 134N | 10W-WT5 |

Number of total and phospho-proteins detected in each run:

|  | Total proteins before NA filtering | Total proteins after NA filtering and merge | Phospho-peptides before NA filtering | Phospho-peptides after NA filtering and merge with totals |
| --- | --- | --- | --- | --- |
| Run 1 | 3998 | 3823 | 6874 | 2792 |
| Run 2 | 4302 |  | 6071 |  |
| Run 3 | 4110 |  | 6780 |  |
| Run 4 | 4597 |  | 7405 |  |

**Kidney** labeling layout:

| **TMT-Run1** |  | **TMT-Run2** |  | **TMT-Run3** |  | **TMT-Run4** |  |
| --- | --- | --- | --- | --- | --- | --- | --- |
| 126 | Pool-1 | 126 | Pool-2 | 126 | Pool-3 | 126 | Pool-4 |
| 127N | 10D-WT1 | ~~127N~~ |  | 127N | 10D KO5 | 127N | 10W KO6 |
| 127C | 10D GS1 | ~~127C~~ |  | 127C | 10D GS+5 | 127C | 10W GS+6 |
| 128N | 10D GS+1 | ~~128N~~ |  | 128N | 10D GS5 | 128N | 10W GS6 |
| 128C | 10D KO1 | 128C | 10W-WT2 | ~~128C~~ |  | 128C | 10W-WT6 |
| 129N | 10W-WT1 | 129N | 10W GS2 | ~~129N~~ |  | 129N | 10D KO6 |
| 129C | 10W GS1 | 129C | 10W GS+2 | ~~129C~~ |  | 129C | 10D GS+6 |
| 130N | 10W GS+1 | 130N | 10W KO2 | 130N | 10D-WT5 | ~~130N~~ |  |
| 130C | 10W KO1 | 130C | 10D-WT3 | 130C | 10W KO4 | ~~130C~~ |  |
| 131N | 10D-WT2 | 131N | 10D GS3 | 131N | 10W GS+4 | ~~131N~~ |  |
| 131C | 10D GS2 | 131C | 10D GS+3 | 131C | 10W GS4 | 131C | 10D GS6 |
| 132N | 10D GS+2 | 132N | 10D KO3 | 132N | 10W-WT4 | 132N | 10D-WT6 |
| 132C | 10D KO2 | 132C | 10W-WT3 | 132C | 10D KO4 | 132C | 10W-KO5 |
| ~~133N~~ |  | 133N | 10W GS3 | 133N | 10D GS+4 | 133N | 10W GS+5 |
| ~~133C~~ |  | 133C | 10W GS+3 | 133C | 10D GS4 | 133C | 10W GS5 |
| ~~134N~~ |  | 134N | 10W KO3 | 134N | 10D-WT4 | 134N | 10W-WT5 |

|  | Total proteins before NA filtering | Total proteins after NA filtering and merge | Phospho-peptides before NA filtering | Phospho-peptides after NA filtering and merge with totals |
| --- | --- | --- | --- | --- |
| Run 1 | 2946 | 2455 | 3226 | 1099 |
| Run 2 | 2920 |  | 3797 |  |
| Run 3 | 3162 |  | 3796 |  |
| Run 4 | 3088 |  | 4104 |  |

**Whole Lung** labeling layout:

| **TMT-Run1** |  | **TMT-Run2** |  |
| --- | --- | --- | --- |
| 126 | Pool-1 | 126 | Pool-2 |
| 127N | 10W-WT_1 | 127N | 10W KO_6 |
| 127C | 10W GS_1 | 127C | 10W GS+_6 |
| 128N | 10W GS+_1 | 128N | 10W GS_6 |
| 128C | 10W KO_1 | 128C | 10W-WT_6 |
| 129N | 10W-WT_2 | 129N | 10W KO_5 |
| 129C | 10W GS_2 | 129C | 10W GS+_5 |
| 130N | 10W GS+_2 | 130N | 10W GS_5 |
| 130C | 10W KO_2 | 130C | 10W-WT_5 |
| 131N | 10W-WT_3 | ~~131N~~ |  |
| 131C | 10W GS_3 | ~~131C~~ |  |
| 132N | 10W GS+_3 | ~~132N~~ |  |
| 132C | 10W KO_3 | 132C | 10W KO_4 |
| ~~133N~~ |  | 133N | 10W GS+_4 |
| ~~133C~~ |  | 133C | 10W GS_4 |
| ~~134N~~ |  | 134N | 10W-WT_4 |

|  | Total proteins before NA filtering | Total proteins after NA filtering and merge | Phospho-peptides before NA filtering | Phospho-peptides after NA filtering and merge with totals |
| --- | --- | --- | --- | --- |
| Run 1 | 3171 | 2702 | 3248 | 911 |
| Run 2 | 3624 |  | 4785 |  |
