# Supplementary material for: Preclinical modeling of chronic inhibition of the Parkinson’s disease associated kinase LRRK2 reveals altered function of the endolysosomal system in vivo
Source: Mol Neurodegener. 2021 Mar 19;16:17. doi: 10.1186/s13024-021-00441-8 (PMC7977595; doi:10.1186/s13024-021-00441-8)
Supplement: Supplementary file 5 — Additional file 5:. [file 13024_2021_441_MOESM5_ESM.pdf]

These membranes were cut according to the molecular weight markers before blotting so that multiple proteins in different molecular-weight ranges could be probed using different strips of the membrane.

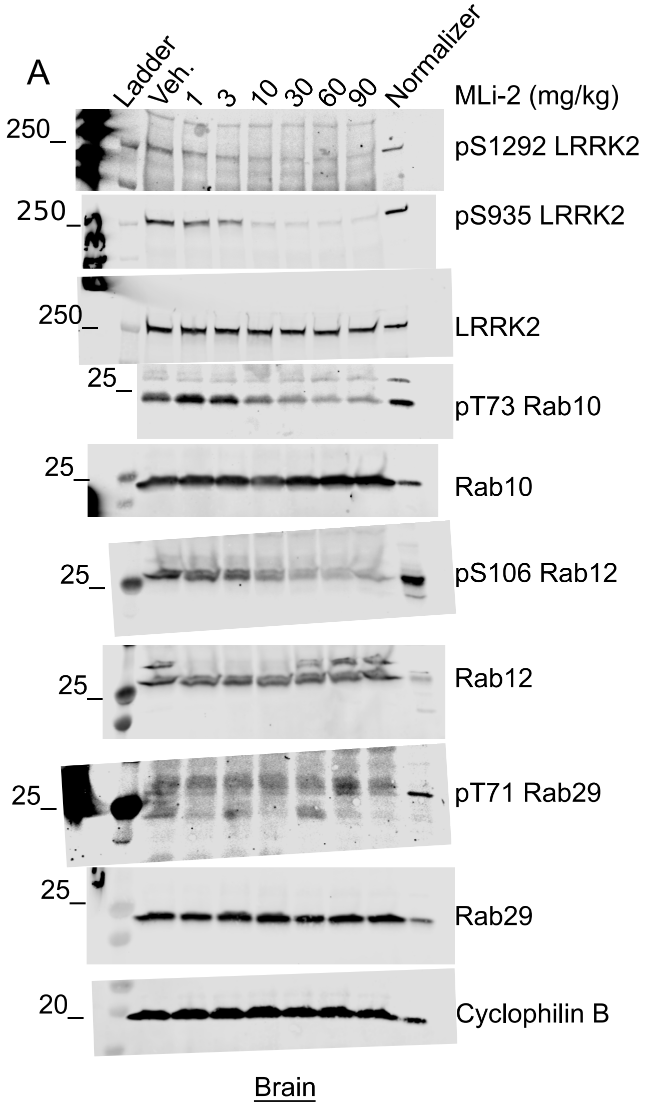

Fig. 1A Brain

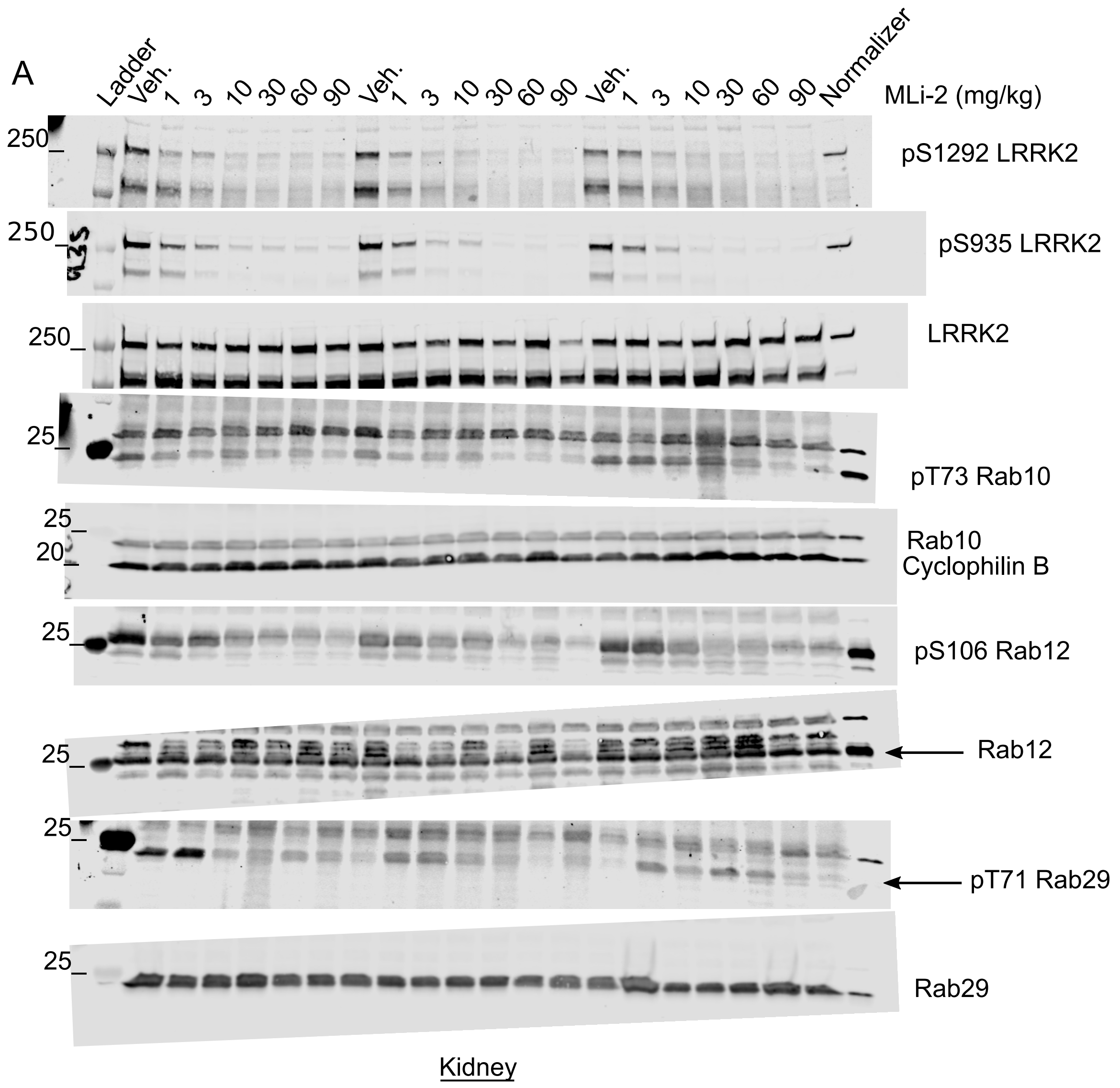

Fig. 1A Kidney

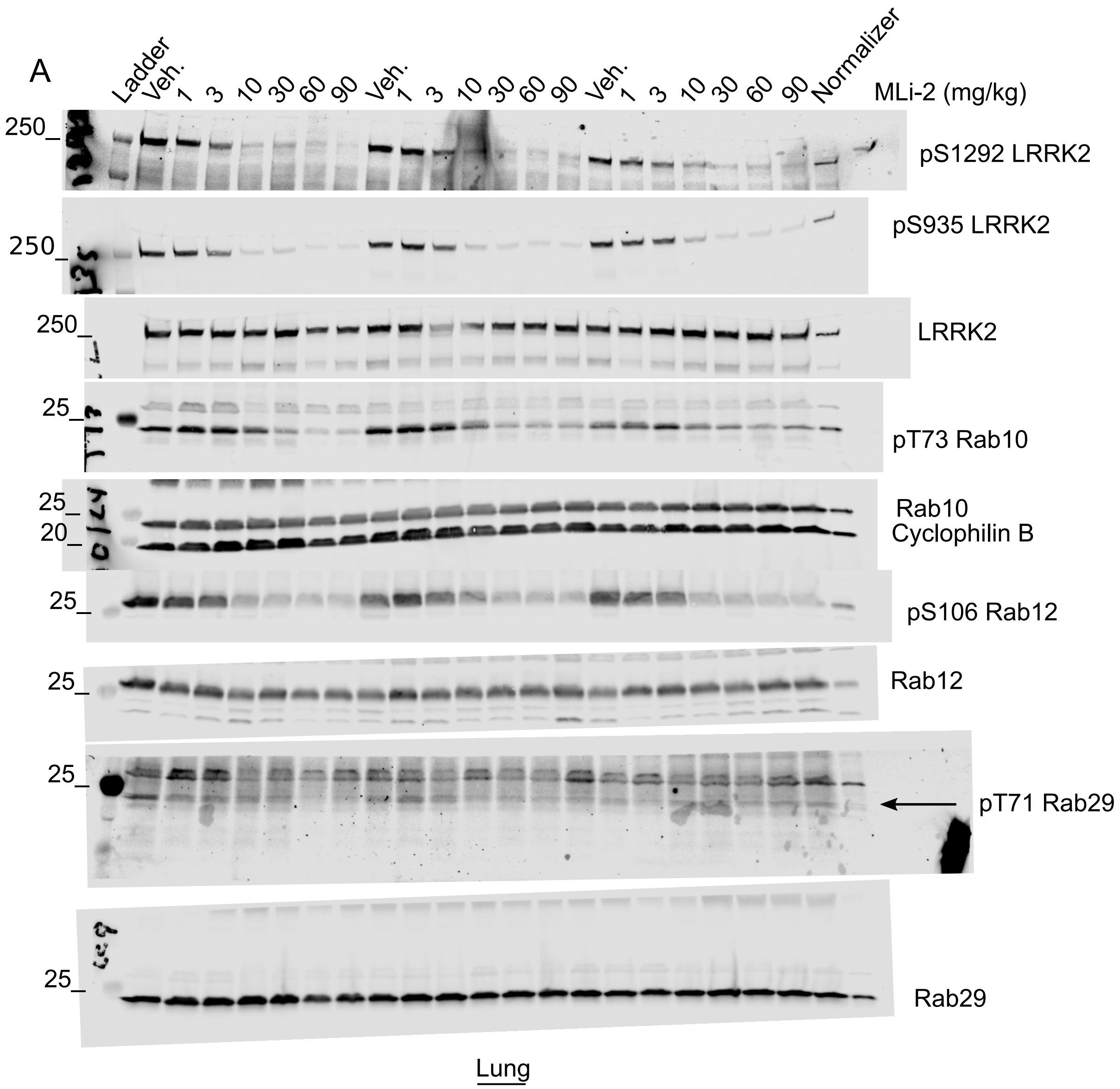
A
Ladder
Veh.
1
3
10
30
60
90
Veh.
1
3
10
30
60
90
Veh.
1
3
10
30
60
90
Normalizer
MLi-2 (mg/kg)
250_
pS1292 LRRK2
250_
pS935 LRRK2
250_
LRRK2
25_
pT73 Rab10
25_
20_
Rab10
Cyclophilin B
25_
pS106 Rab12
25_
Rab12
25_
pT71 Rab29
25_
Rab29
Lung

Fig. 1A Lung

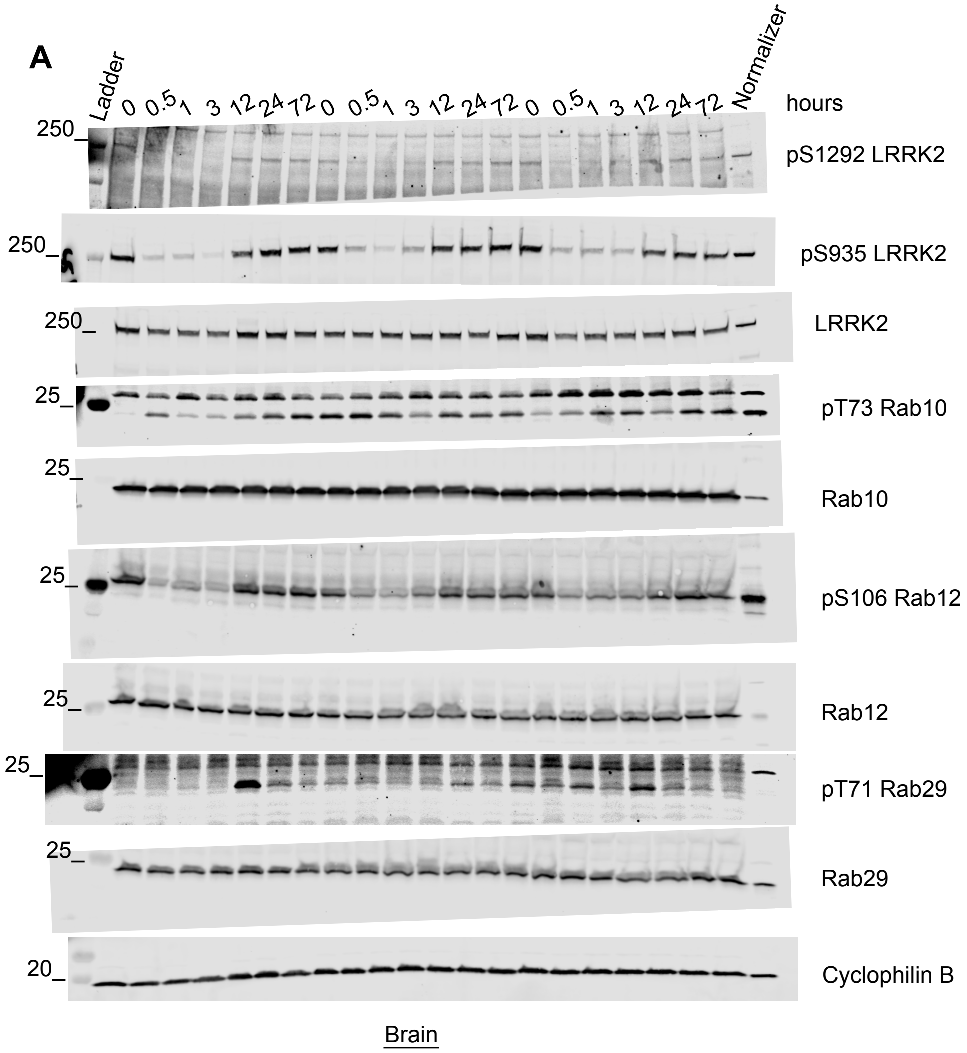
A
Ladder
0 0.5 1 3 12 24 72 0 0.5 1 3 12 24 72 0 0.5 1 3 12 24 72
Normalizer
hours
250_
pS1292 LRRK2
250_
pS935 LRRK2
250_
LRRK2
25_
pT73 Rab10
25_
Rab10
25_
pS106 Rab12
25_
Rab12
25_
pT71 Rab29
25_
Rab29
20_
Cyclophilin B
Brain

Fig. 2A Brain

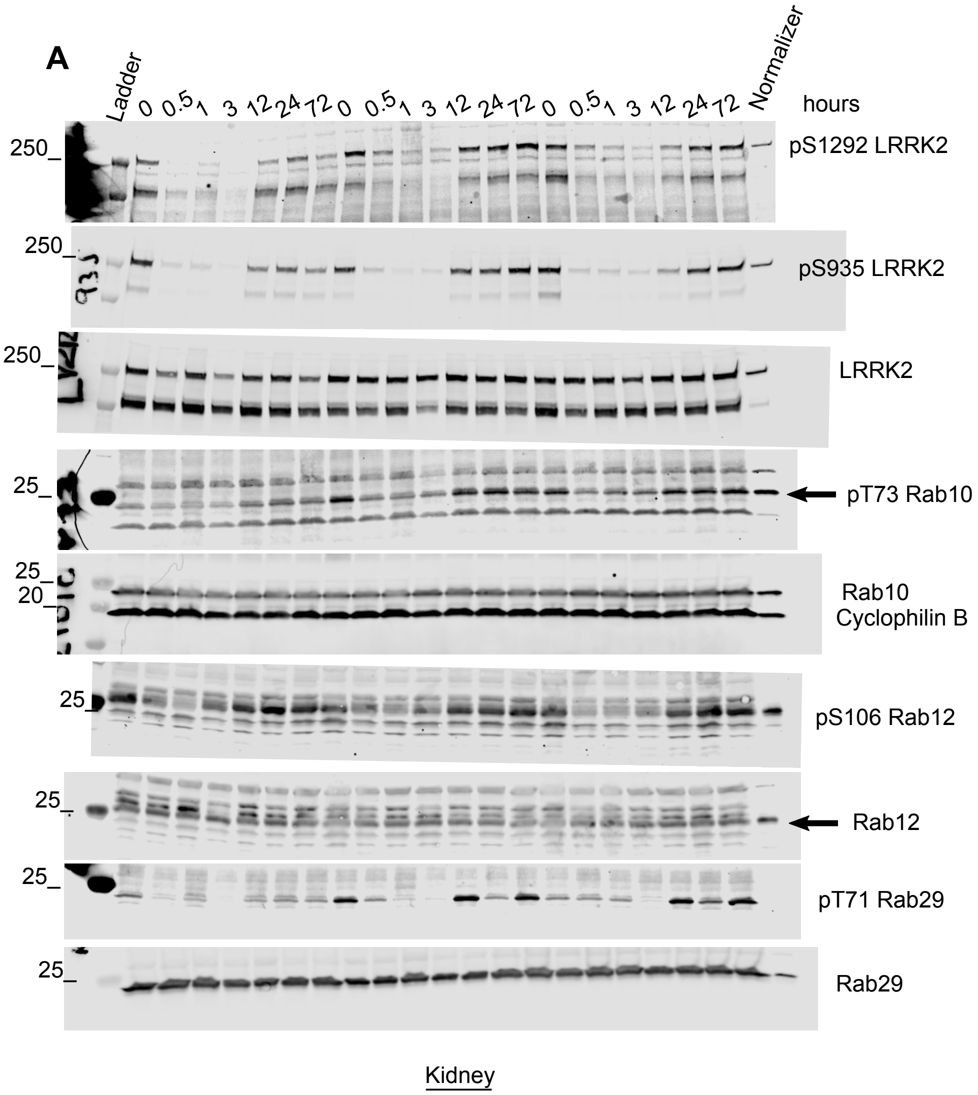

Fig. 2A Kidney

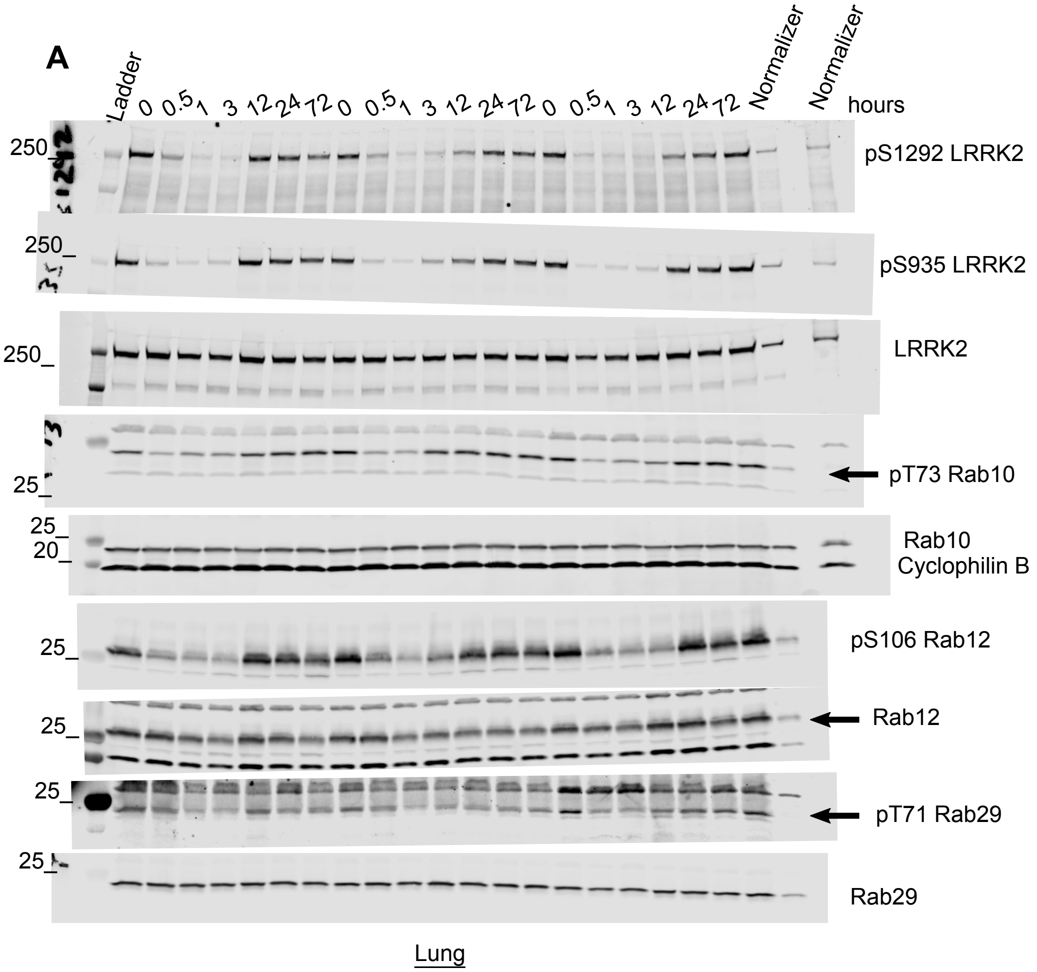
A
Ladder
0
0.5
1
3
12
24
72
Normalizer
Normalizer
hours
250
pS1292 LRRK2
pS935 LRRK2
LRRK2
25
pT73 Rab10
20
Rab10
Cyclophilin B
pS106 Rab12
Rab12
pT71 Rab29
Rab29
Lung

Fig. 2A Lung

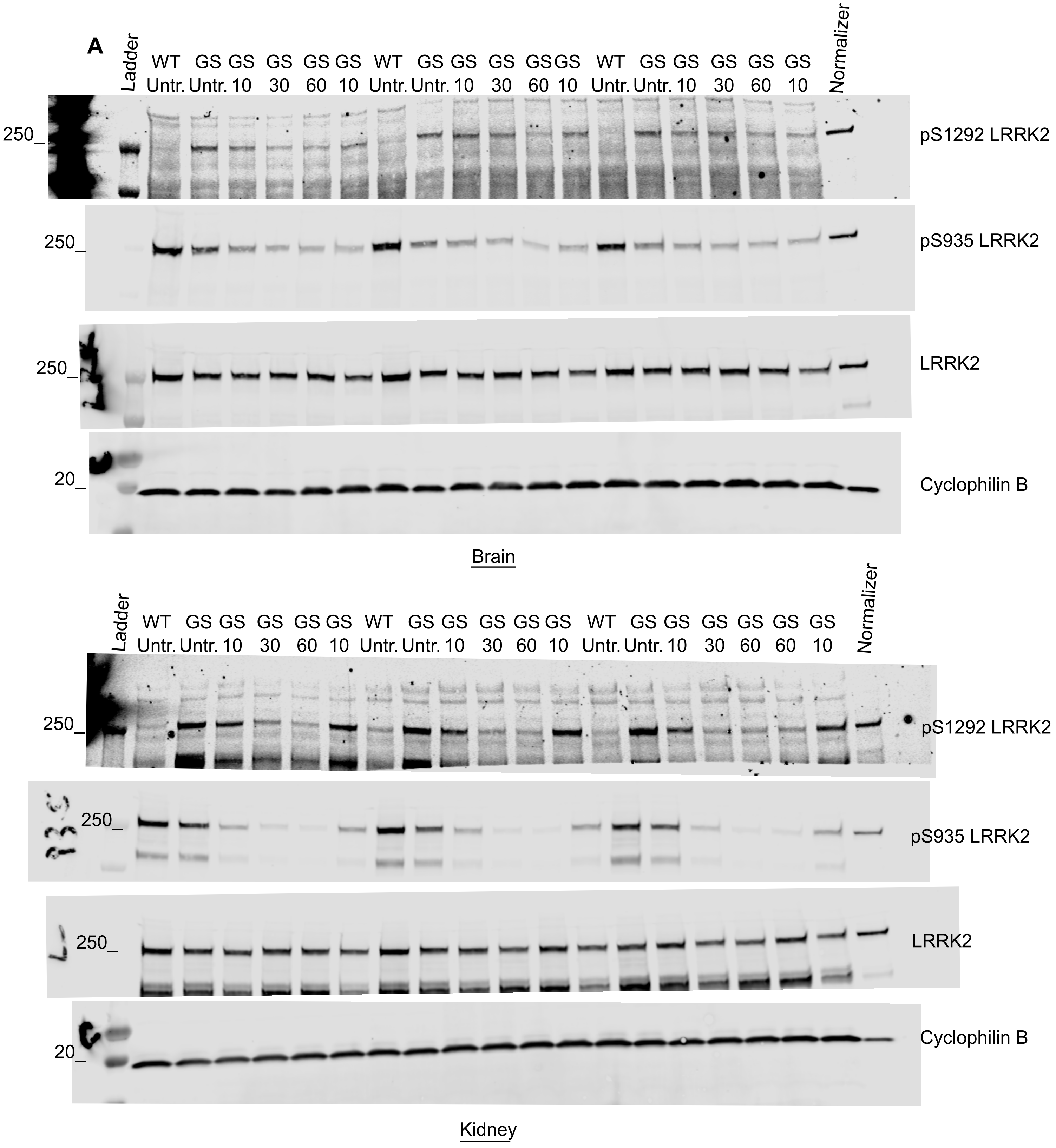

Fig. 3A Brain and Kidney

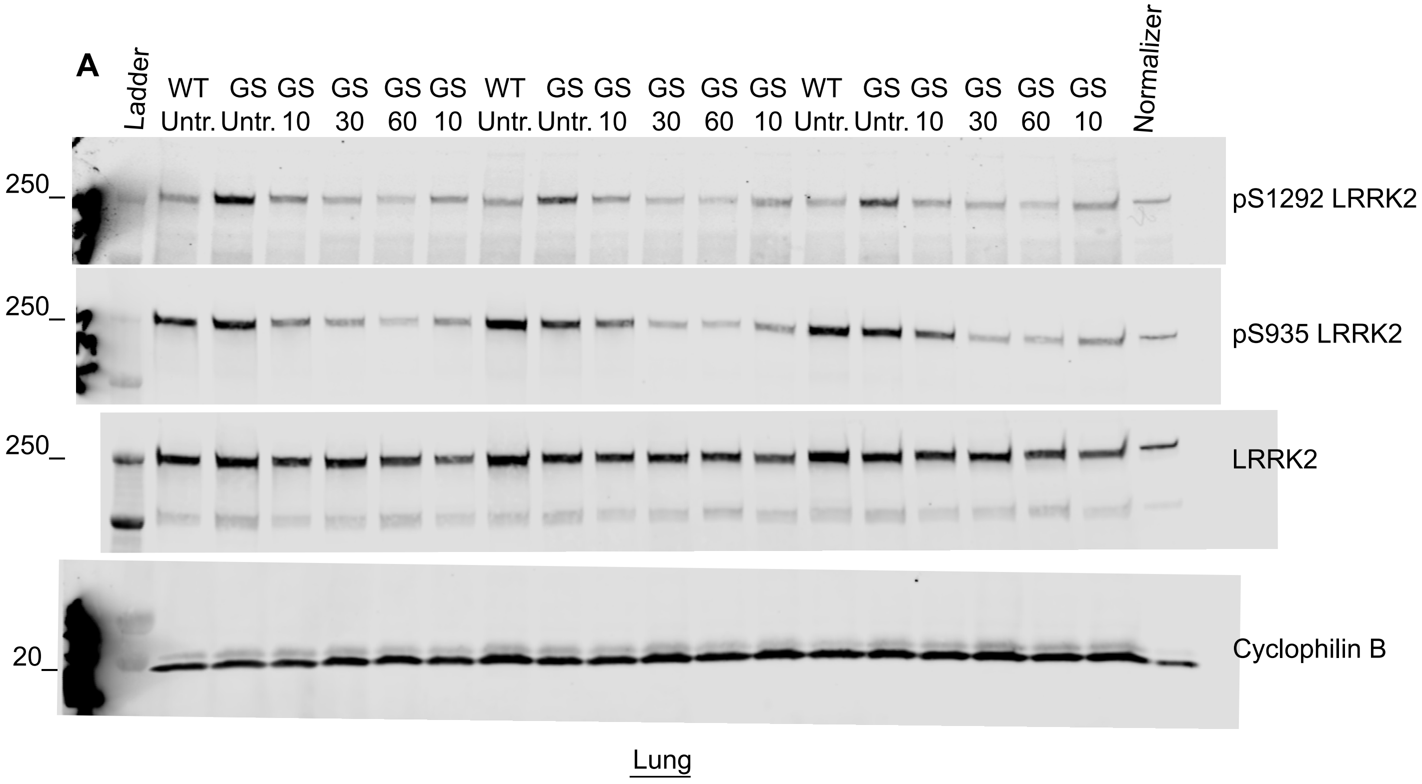
A
Ladder
WT Untr.
GS Untr.
GS 10
GS 30
GS 60
GS 10
WT Untr.
GS Untr.
GS 10
GS 30
GS 60
GS 10
WT Untr.
GS Untr.
GS 10
GS 30
GS 60
GS 10
Normalizer
250_
pS1292 LRRK2
250_
pS935 LRRK2
250_
LRRK2
20_
Cyclophilin B
Lung

Fig. 3A Lung

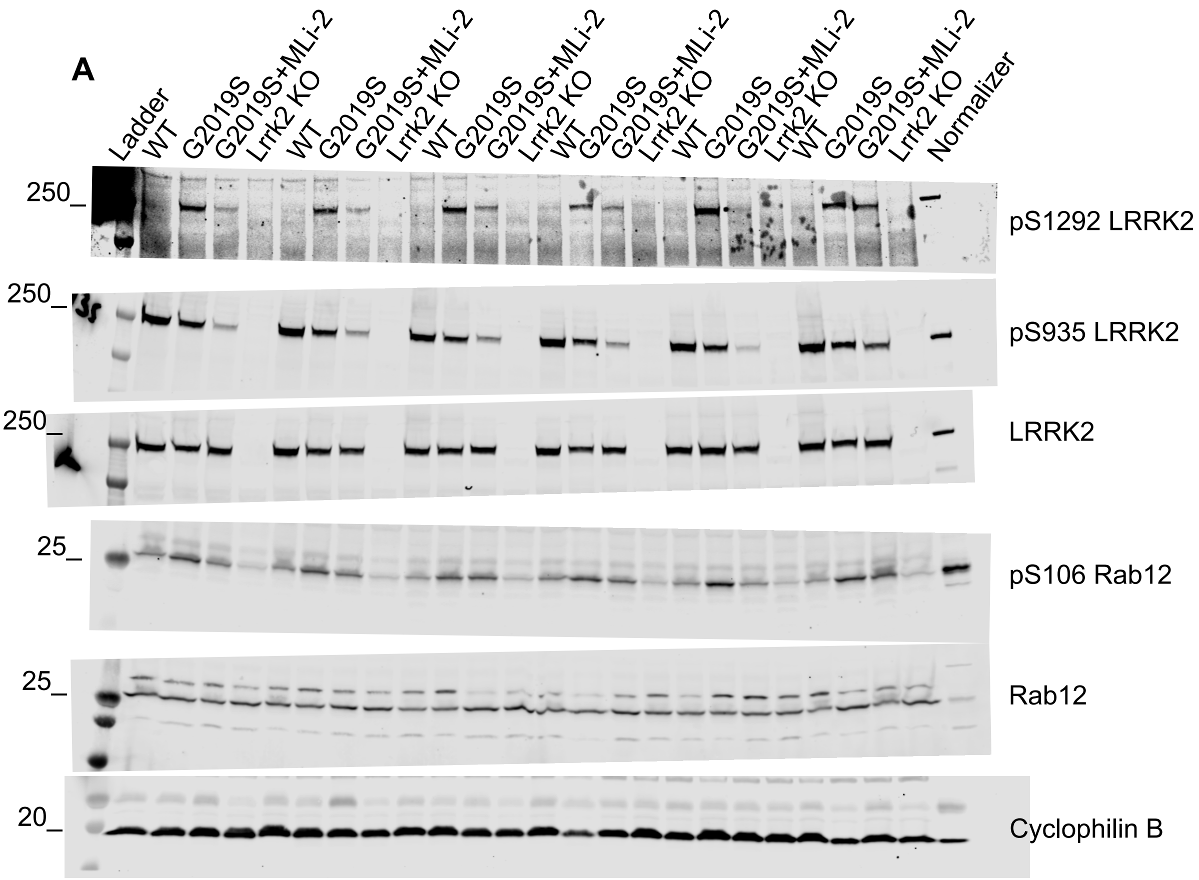

Brain

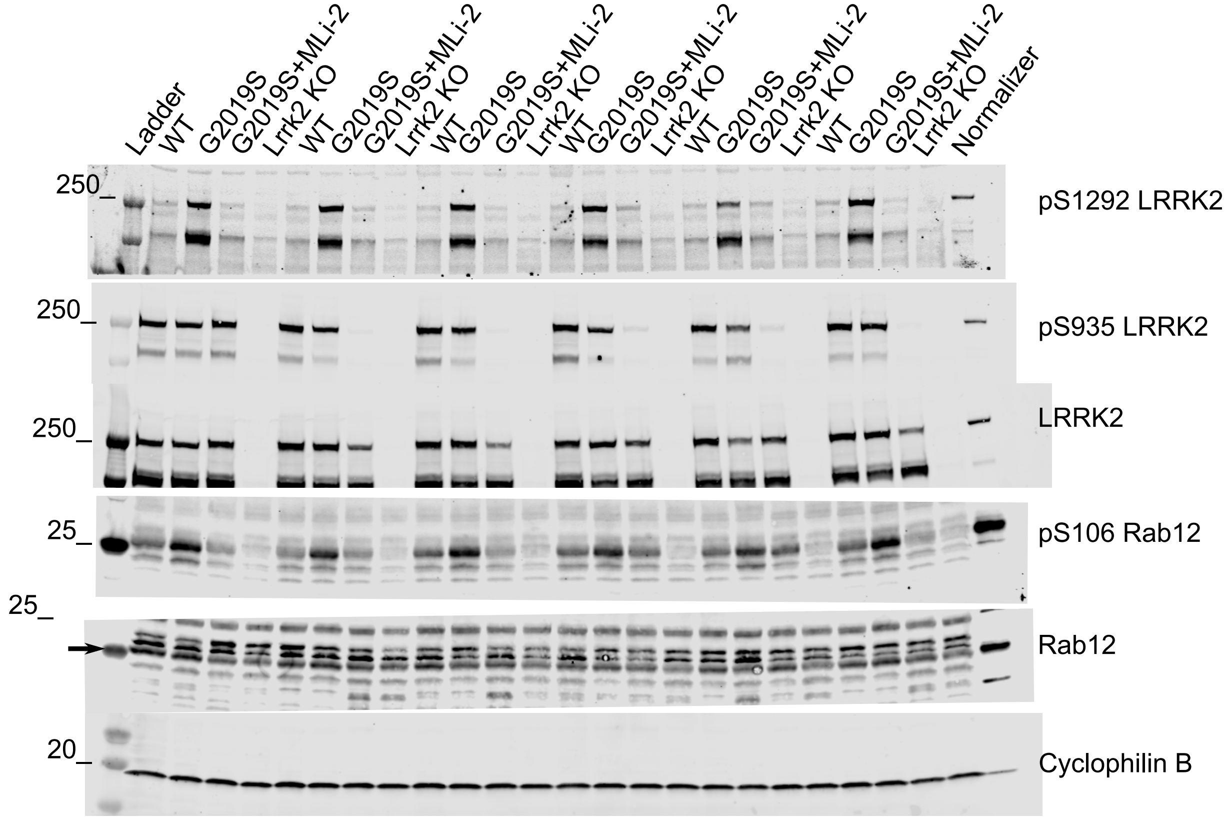

Kidney

10 Day Cohort

Fig. 5A Brain and Kidney 10 days

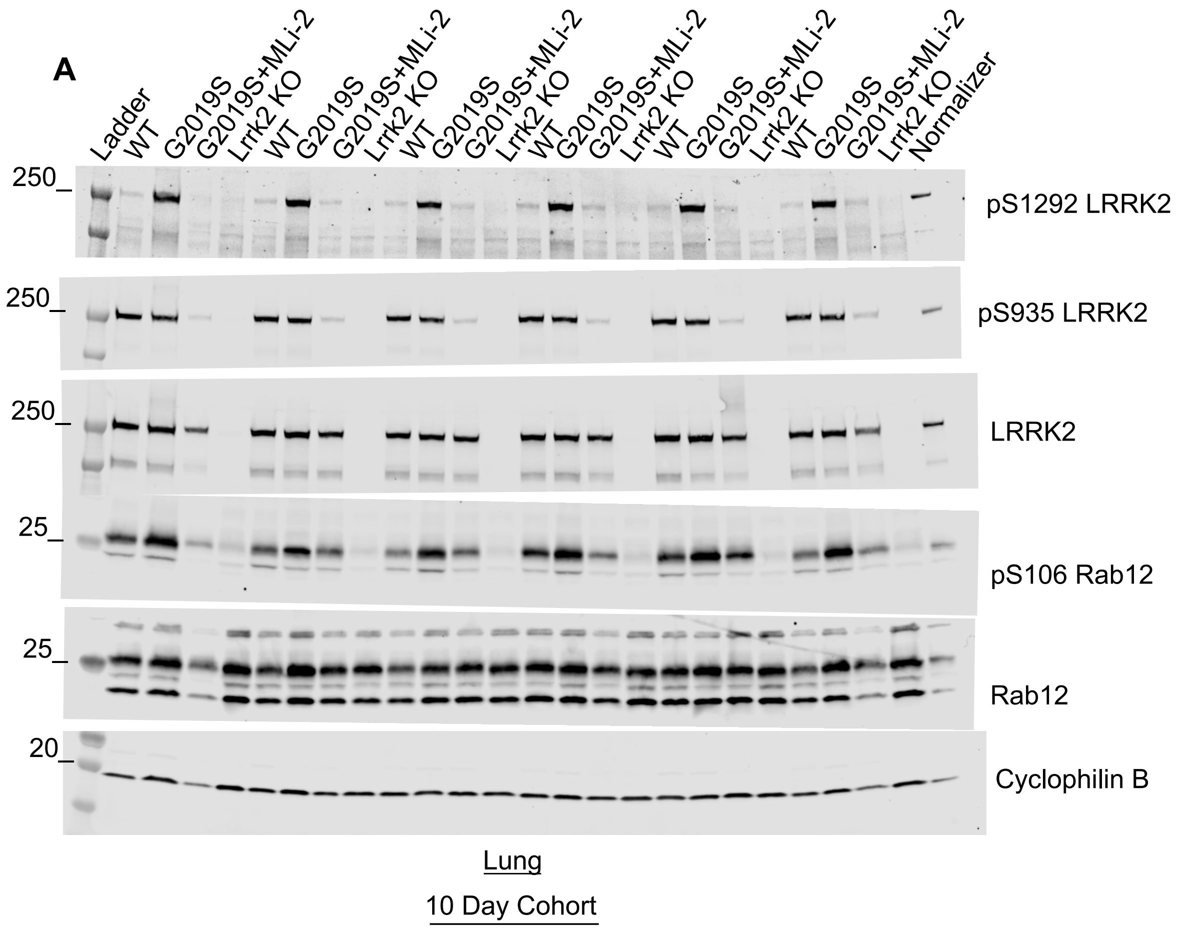

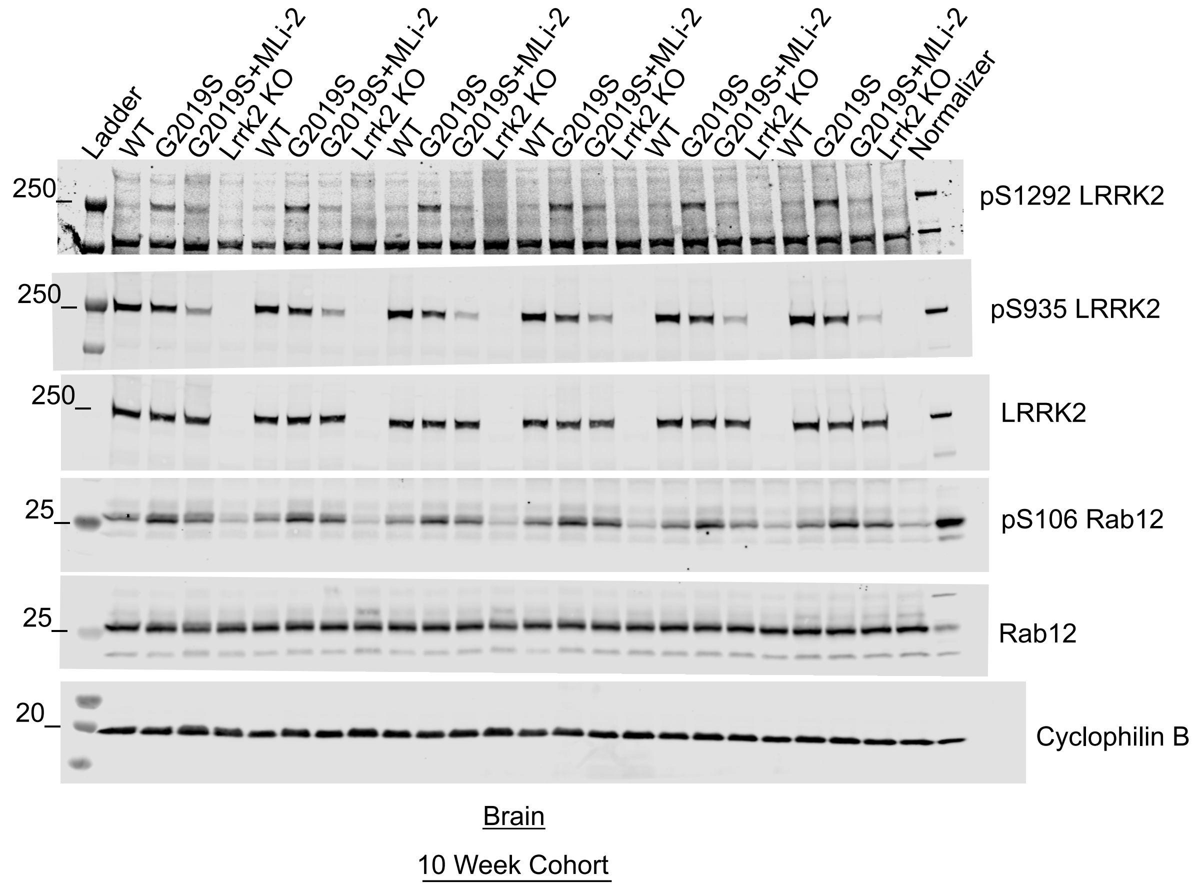

Fig. 5A Lung days and Brain weeks

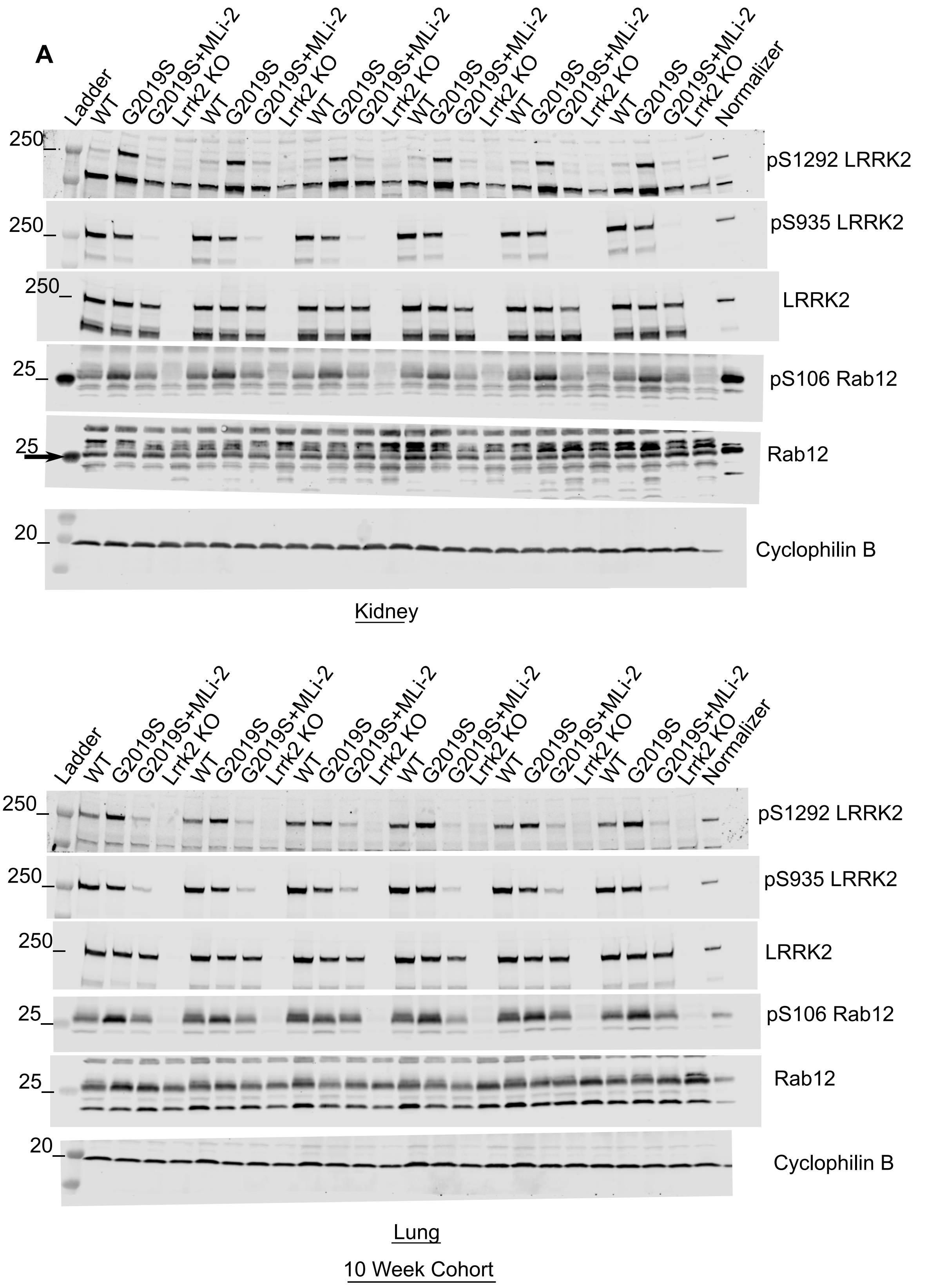

Fig. 5A Kidney and Lung weeks

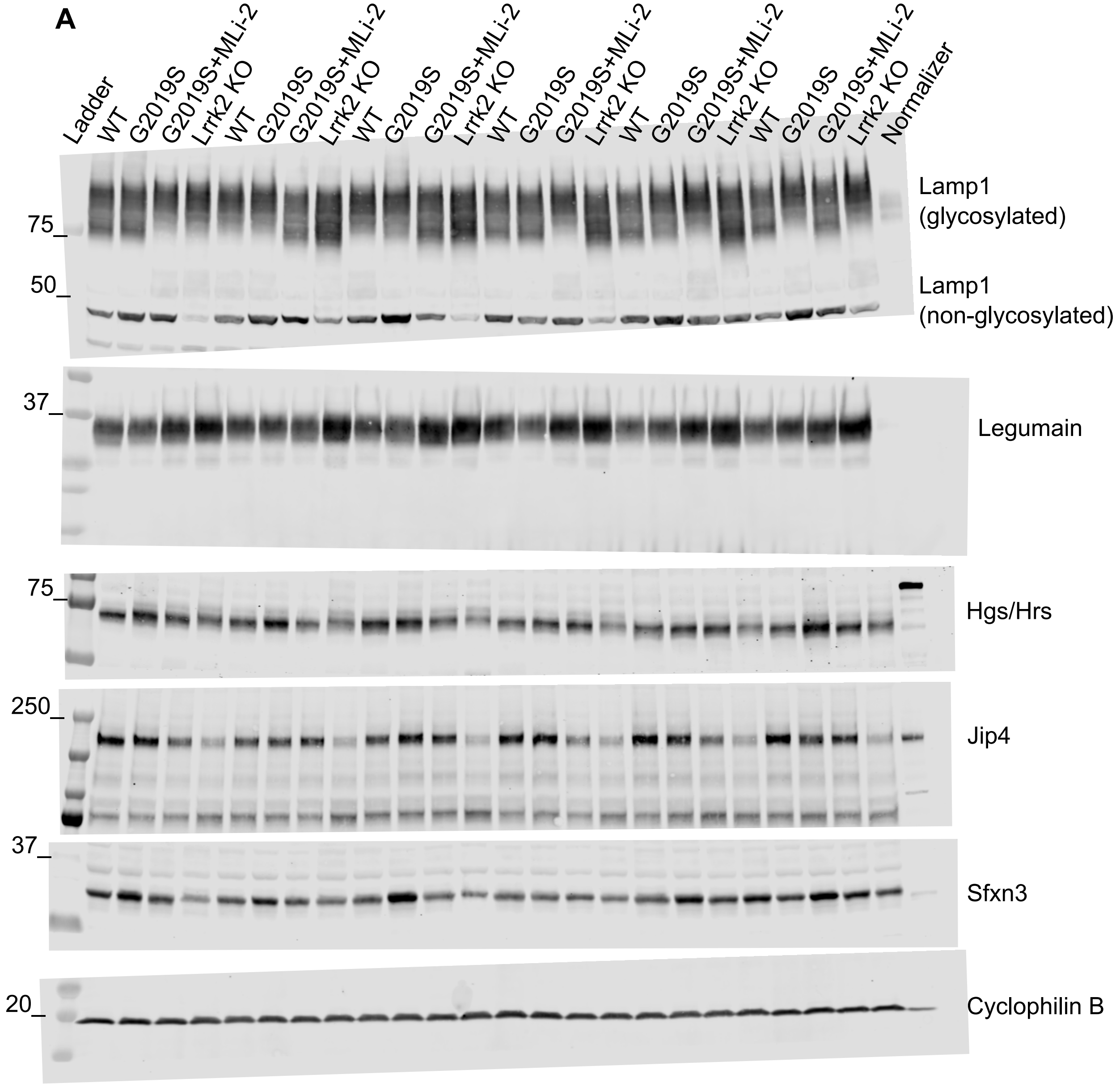
A
Ladder
WT
G2019S
G2019S+MLi-2
Lrrk2 KO
WT
G2019S
G2019S+MLi-2
Lrrk2 KO
WT
G2019S
G2019S+MLi-2
Lrrk2 KO
WT
G2019S
G2019S+MLi-2
Lrrk2 KO
WT
G2019S
G2019S+MLi-2
Lrrk2 KO
WT
G2019S
G2019S+MLi-2
Lrrk2 KO
Normalizer
75_
50_
Lamp1 (glycosylated)
Lamp1 (non-glycosylated)
37_
Legumain
75_
Hgs/Hrs
250_
Jip4
37_
Sfxn3
20_
Cyclophilin B

Fig. 8A

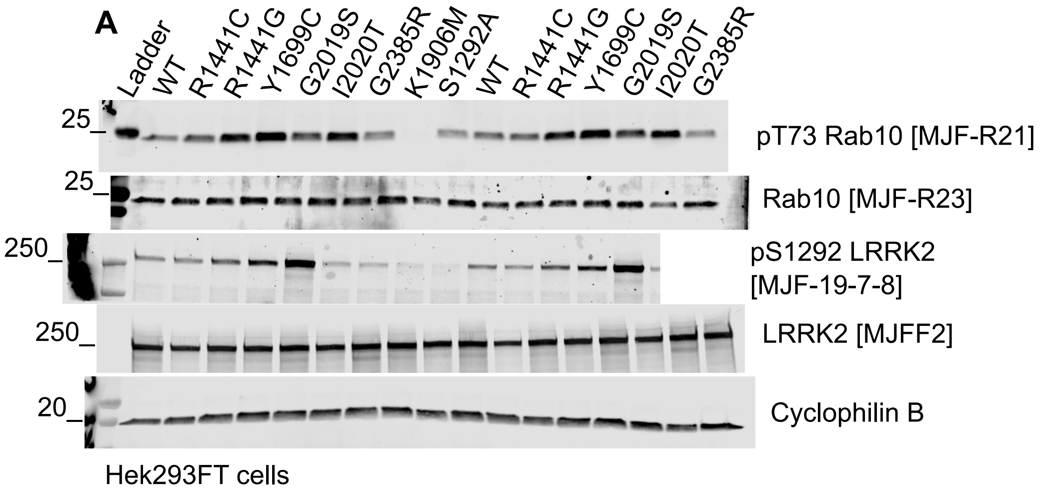
A
Ladder
WT
R1441C
R1441G
Y1699C
G2019S
I2020T
G2385R
K1906M
S1292A
WT
R1441C
R1441G
Y1699C
G2019S
I2020T
G2385R
25_
pT73 Rab10 [MJF-R21]
25_
Rab10 [MJF-R23]
250_
pS1292 LRRK2 [MJF-19-7-8]
250_
LRRK2 [MJFF2]
20_
Cyclophilin B
Hek293FT cells

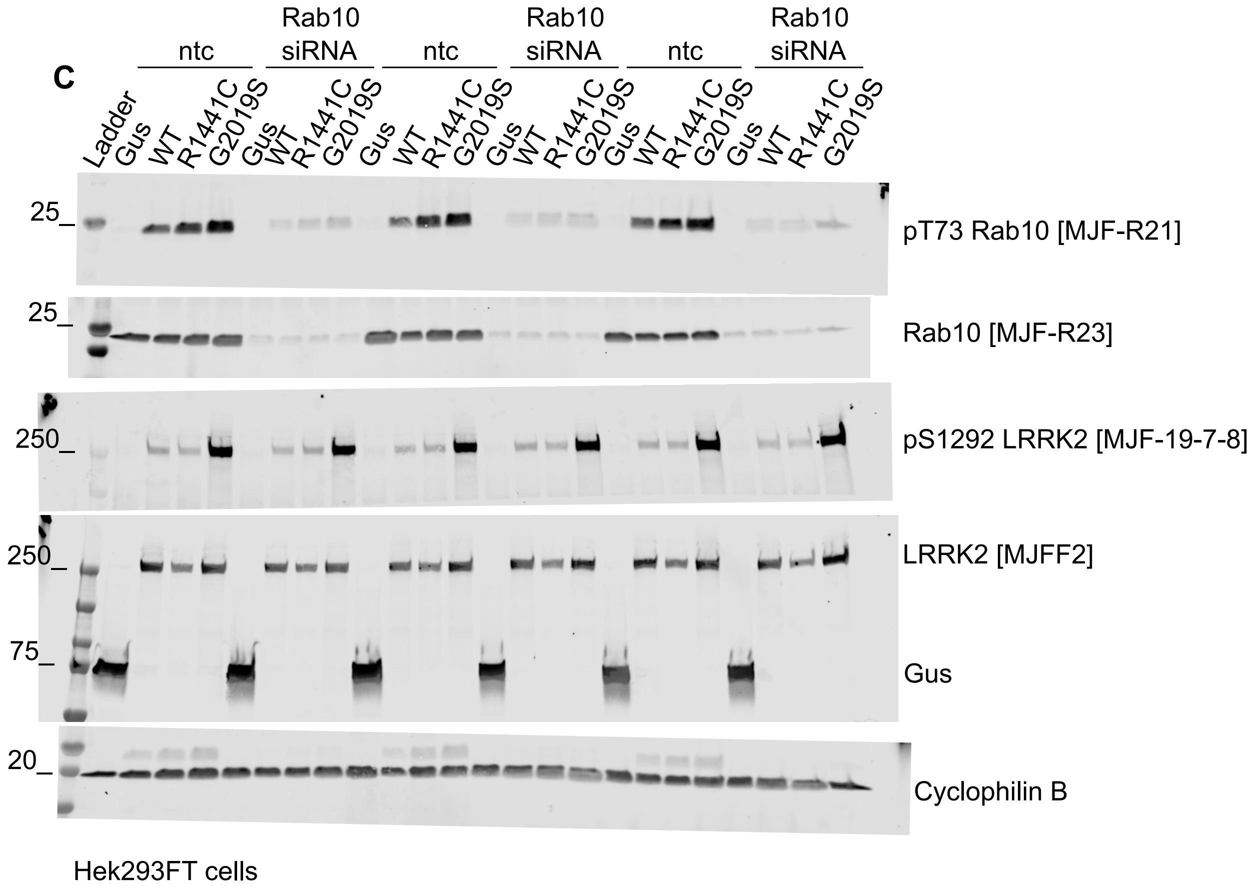
C
ntc
Rab10 siRNA
ntc
Rab10 siRNA
ntc
Rab10 siRNA
Ladder
Gus
WT
R1441C
G2019S
Gus
WT
R1441C
G2019S
Gus
WT
R1441C
G2019S
Gus
WT
R1441C
G2019S
Gus
WT
R1441C
G2019S
Gus
WT
R1441C
G2019S
25_
pT73 Rab10 [MJF-R21]
25_
Rab10 [MJF-R23]
250_
pS1292 LRRK2 [MJF-19-7-8]
250_
LRRK2 [MJFF2]
75_
Gus
20_
Cyclophilin B
Hek293FT cells

Fig. S2A, C

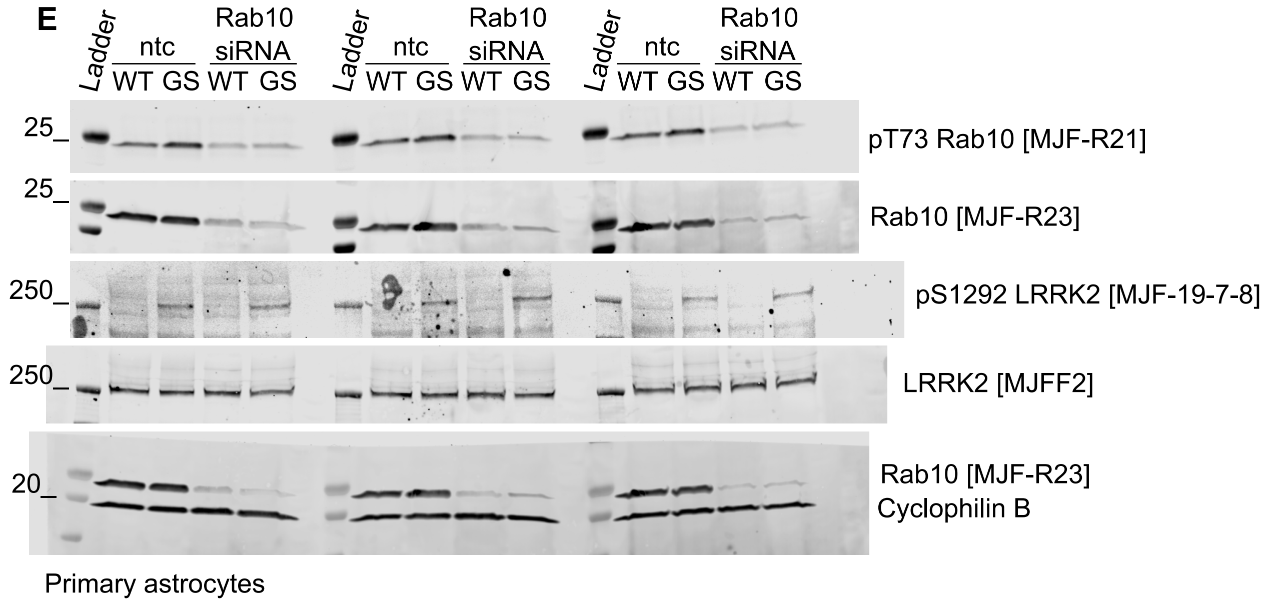
E
Ladder
ntc
Rab10 siRNA
WT GS WT GS
25_
pT73 Rab10 [MJF-R21]
25_
Rab10 [MJF-R23]
250_
pS1292 LRRK2 [MJF-19-7-8]
250_
LRRK2 [MJFF2]
20_
Rab10 [MJF-R23]
Cyclophilin B
Primary astrocytes

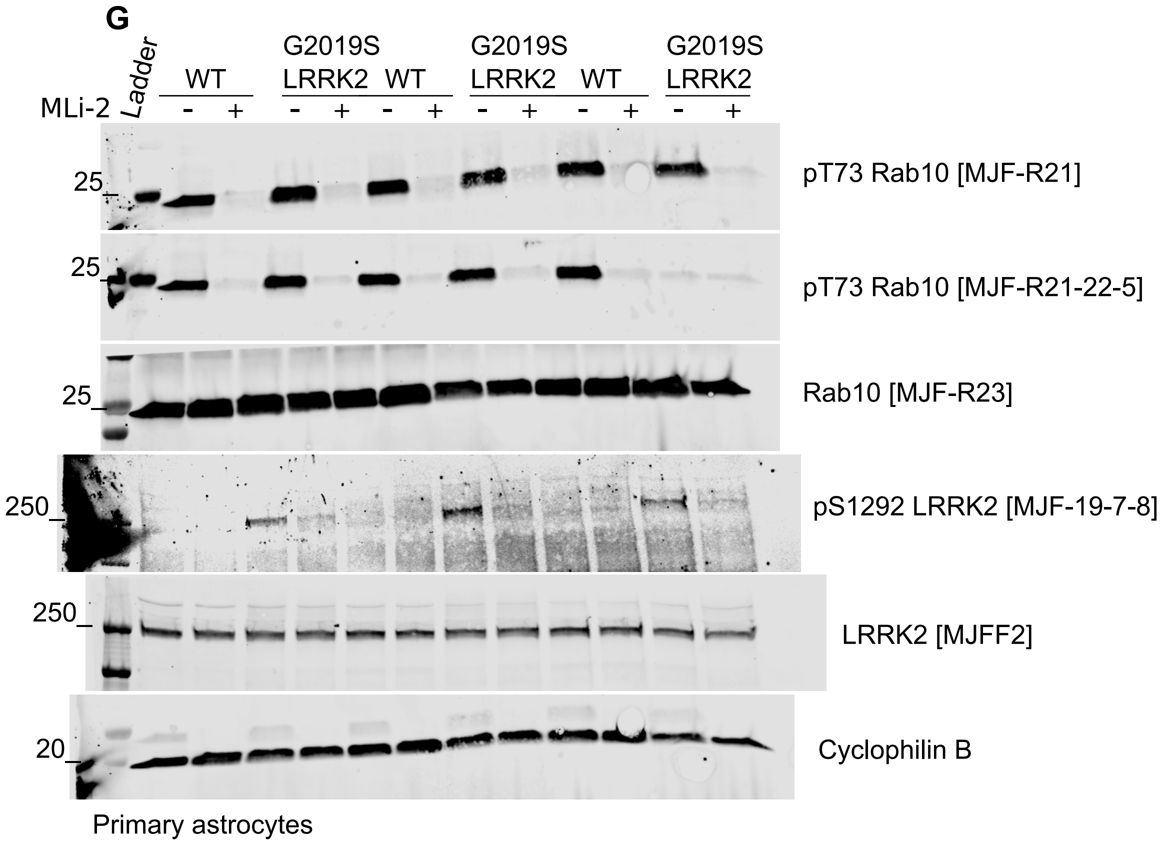
G
Ladder
WT
G2019S LRRK2
MLi-2
- +
25_
pT73 Rab10 [MJF-R21]
25_
pT73 Rab10 [MJF-R21-22-5]
25_
Rab10 [MJF-R23]
250_
pS1292 LRRK2 [MJF-19-7-8]
250_
LRRK2 [MJFF2]
20_
Cyclophilin B
Primary astrocytes

Fig. S2E, G

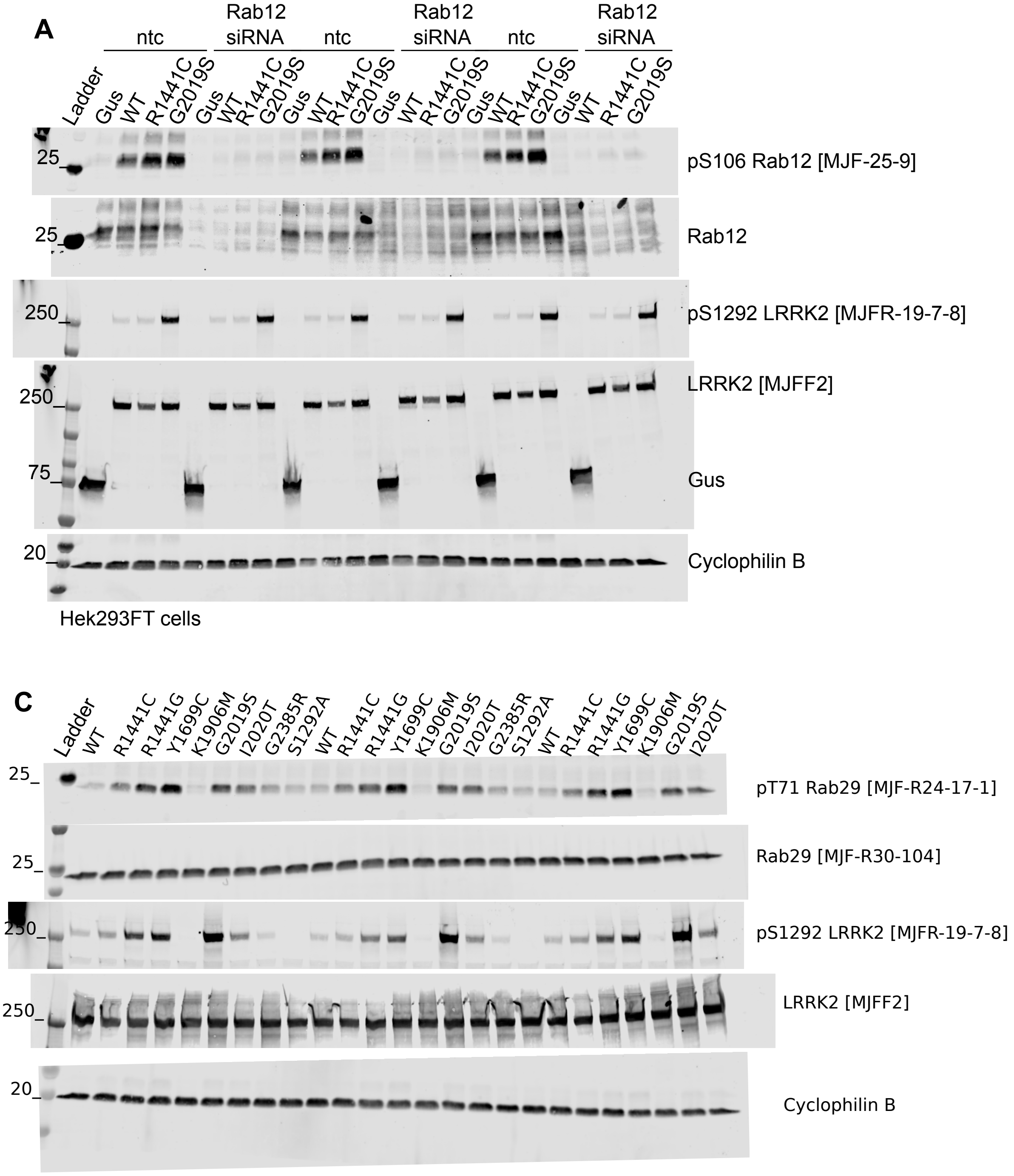
A
ntc
Rab12 siRNA
ntc
Rab12 siRNA
ntc
Rab12 siRNA
Ladder
Gus
WT
R1441C
G2019S
25
250
75
20
pS106 Rab12 [MJF-25-9]
Rab12
pS1292 LRRK2 [MJFR-19-7-8]
LRRK2 [MJFF2]
Gus
Cyclophilin B
Hek293FT cells
C
Ladder
WT
R1441C
R1441G
Y1699C
K1906M
G2019S
I2020T
G2385R
S1292A
pT71 Rab29 [MJF-R24-17-1]
Rab29 [MJF-R30-104]
pS1292 LRRK2 [MJFR-19-7-8]
LRRK2 [MJFF2]
Cyclophilin B

Fig. S3A, C

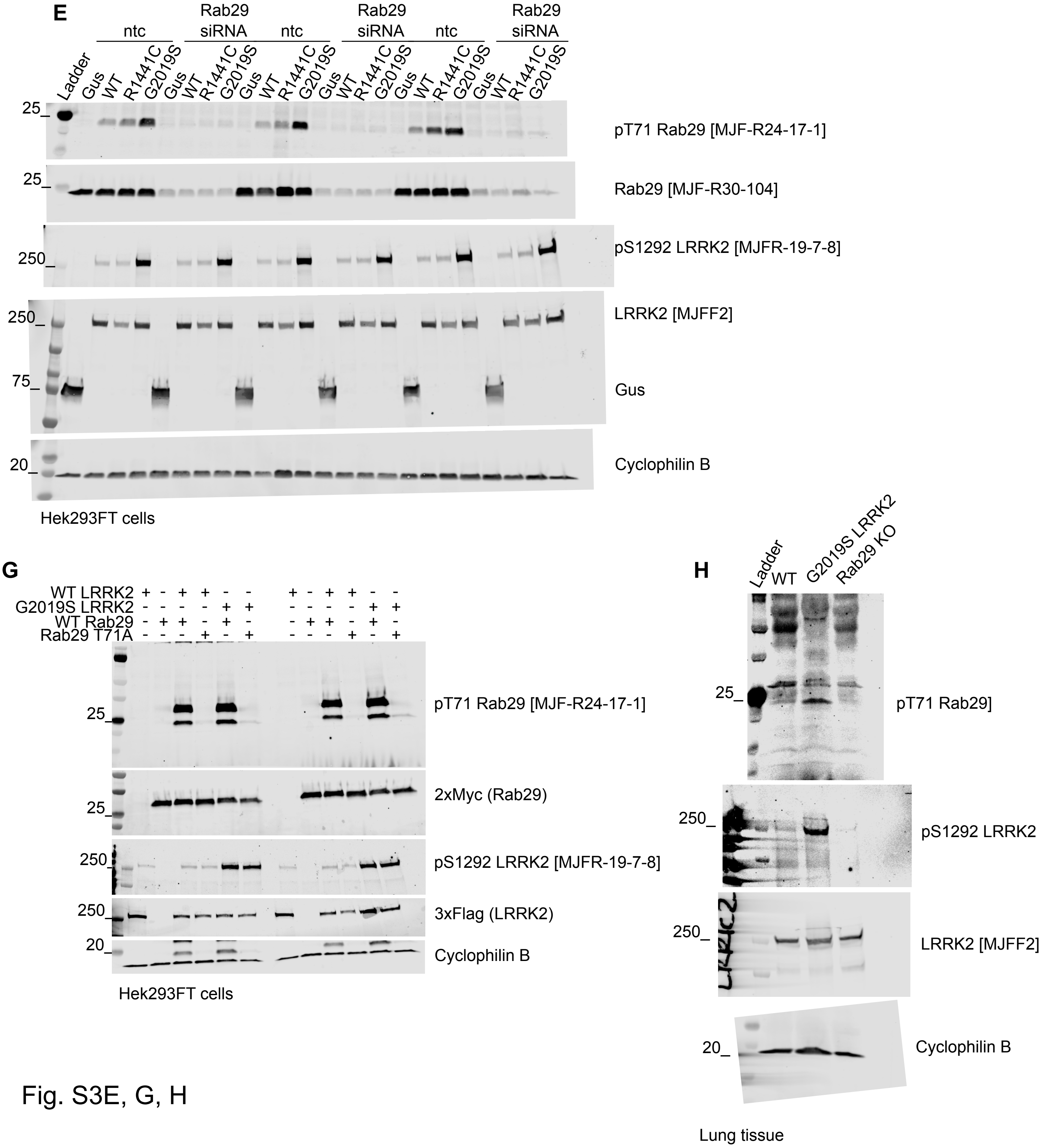
E
ntc
Rab29 siRNA
ntc
Rab29 siRNA
ntc
Rab29 siRNA
Ladder
Gus
WT
R1441C
G2019S
25_
pT71 Rab29 [MJF-R24-17-1]
25_
Rab29 [MJF-R30-104]
250_
pS1292 LRRK2 [MJFR-19-7-8]
250_
LRRK2 [MJFF2]
75_
Gus
20_
Cyclophilin B
Hek293FT cells
G
WT LRRK2
G2019S LRRK2
WT Rab29
Rab29 T71A
25_
pT71 Rab29 [MJF-R24-17-1]
25_
2xMyc (Rab29)
250_
pS1292 LRRK2 [MJFR-19-7-8]
250_
3xFlag (LRRK2)
20_
Cyclophilin B
Hek293FT cells
H
Ladder
WT
G2019S LRRK2
Rab29 KO
25_
pT71 Rab29]
250_
pS1292 LRRK2
250_
LRRK2 [MJFF2]
20_
Cyclophilin B
Lung tissue

Fig. S3E, G, H

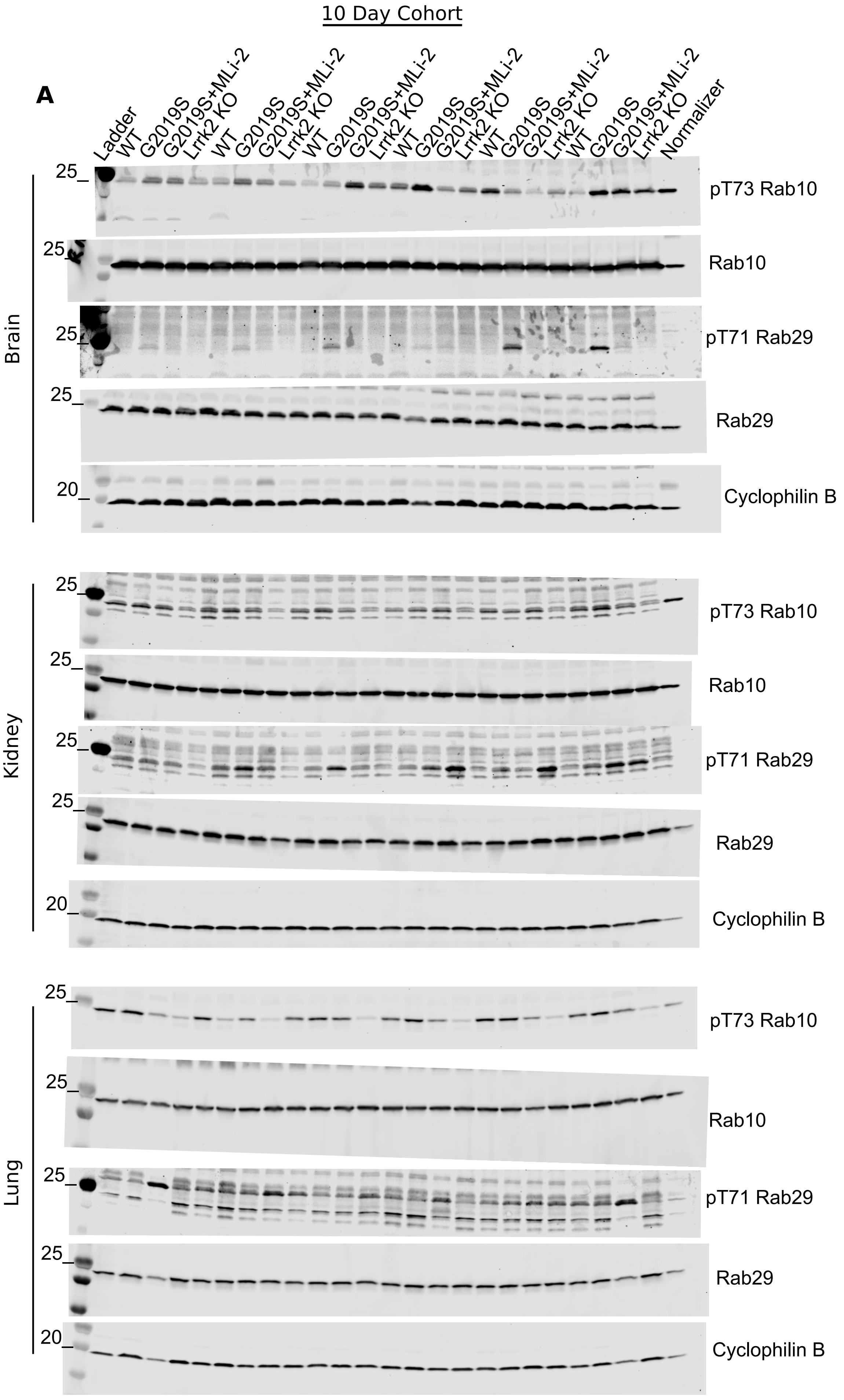
10 Day Cohort
A
Ladder
WT
G2019S
G2019S+MLi-2
Lrrk2 KO
Normalizer
Brain
Kidney
Lung
pT73 Rab10
Rab10
pT71 Rab29
Rab29
Cyclophilin B
25
20

Fig. S4A Days

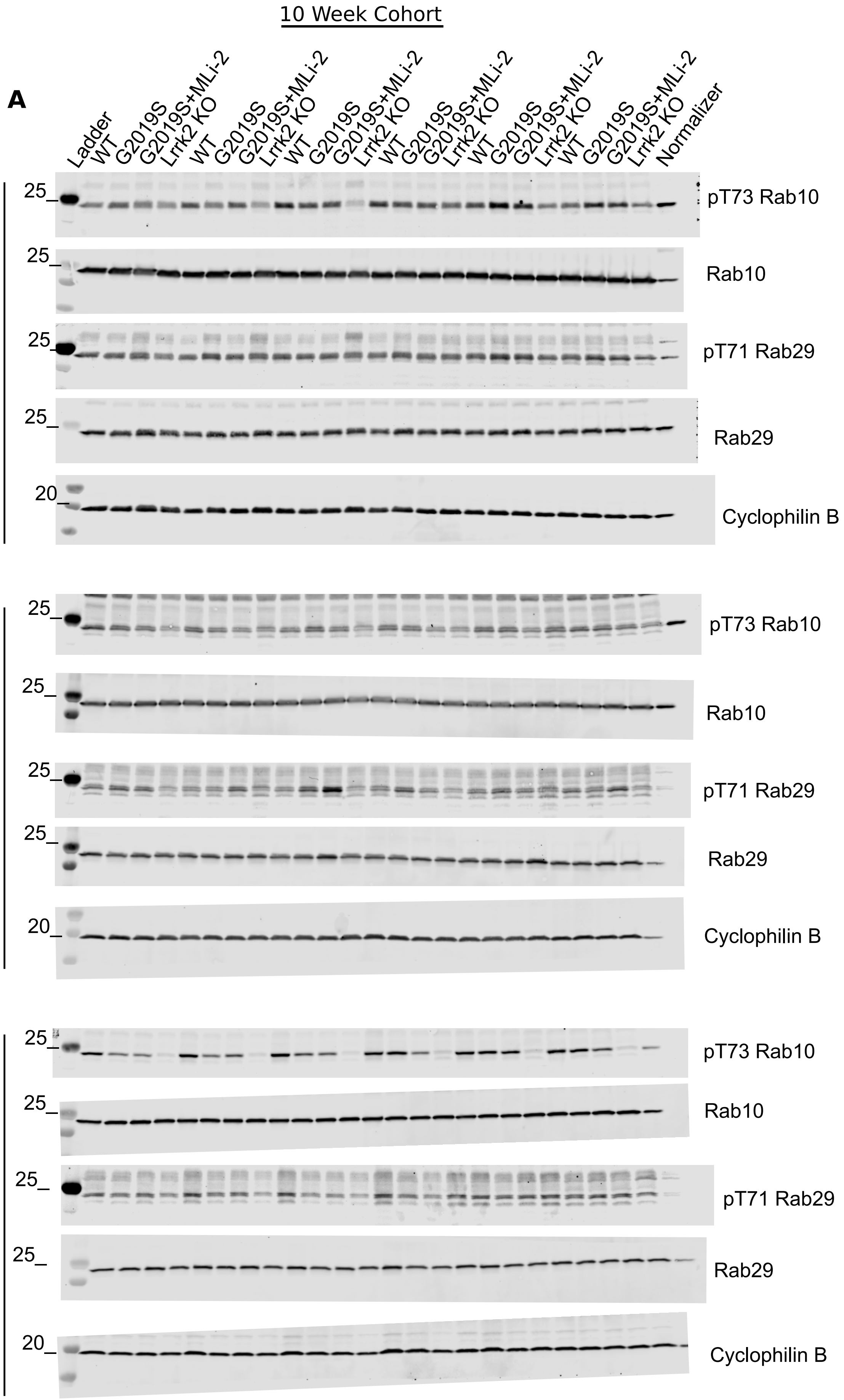

Fig. S4A Weeks

A

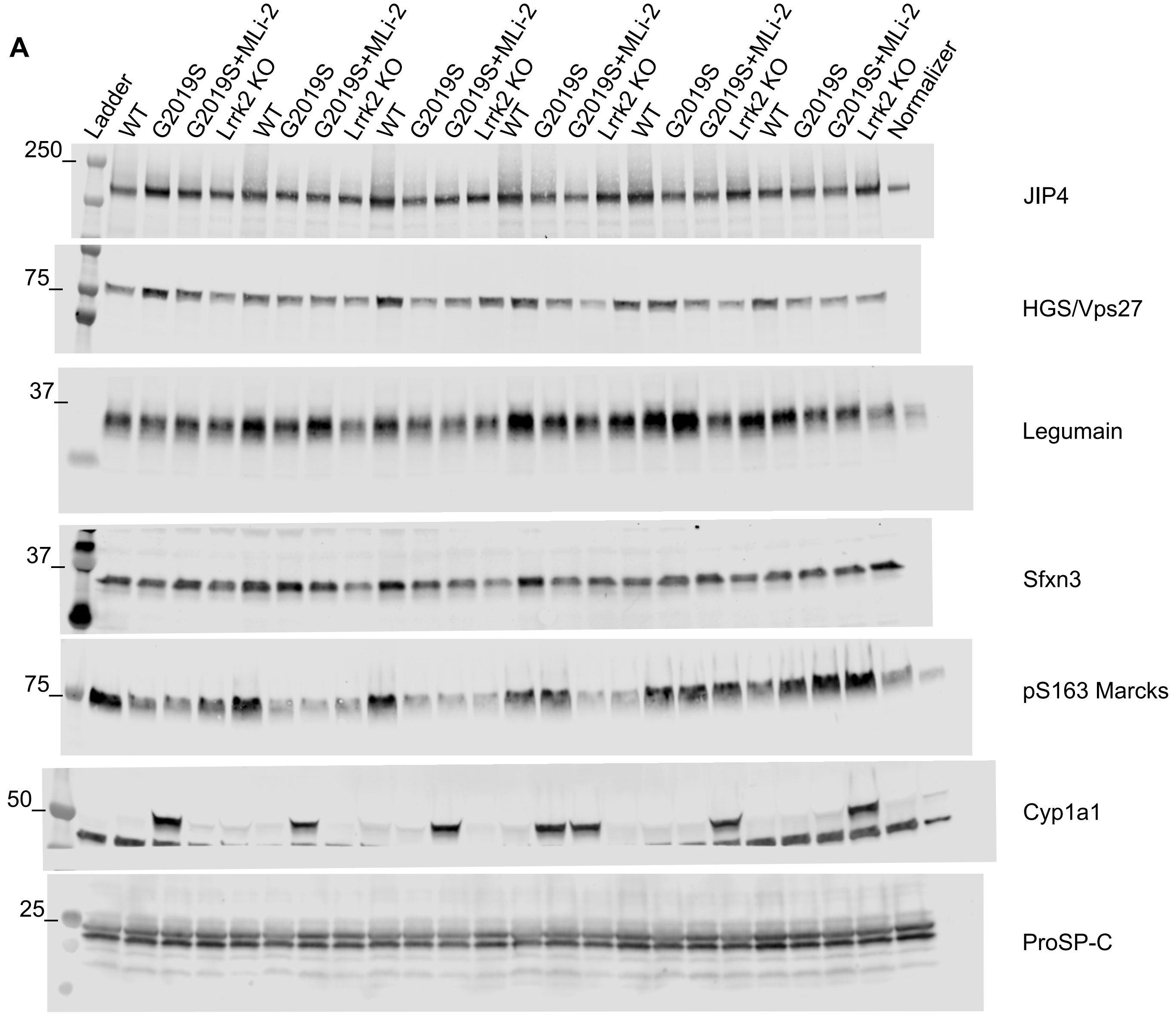
Ladder
WT
G2019S
G2019S+MLi-2
Lrrk2 KO
WT
G2019S
G2019S+MLi-2
Lrrk2 KO
WT
G2019S
G2019S+MLi-2
Lrrk2 KO
WT
G2019S
G2019S+MLi-2
Lrrk2 KO
WT
G2019S
G2019S+MLi-2
Lrrk2 KO
WT
G2019S
G2019S+MLi-2
Lrrk2 KO
Normalizer
250
JIP4
75
HGS/Vps27
37
Legumain
37
Sfxn3
75
pS163 Marcks
50
Cyp1a1
25
ProSP-C

C

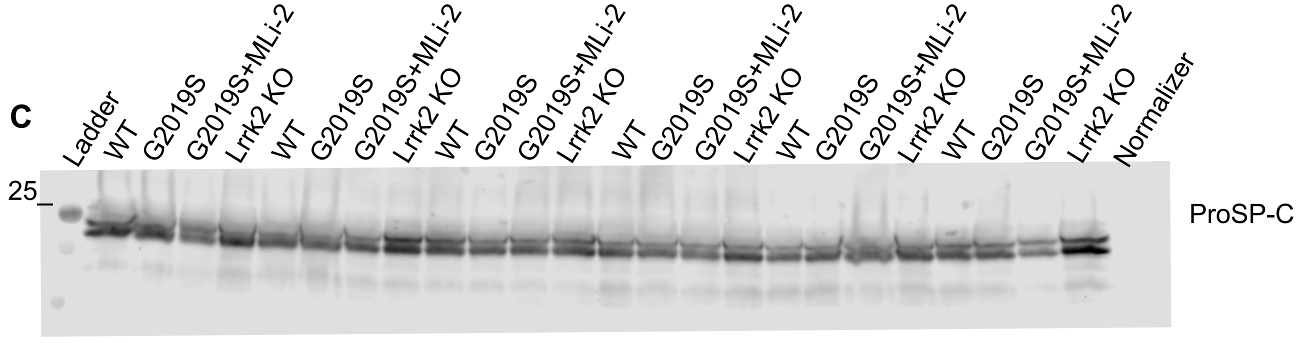
Ladder
WT
G2019S
G2019S+MLi-2
Lrrk2 KO
WT
G2019S
G2019S+MLi-2
Lrrk2 KO
WT
G2019S
G2019S+MLi-2
Lrrk2 KO
WT
G2019S
G2019S+MLi-2
Lrrk2 KO
WT
G2019S
G2019S+MLi-2
Lrrk2 KO
WT
G2019S
G2019S+MLi-2
Lrrk2 KO
Normalizer
25
ProSP-C

Fig. S6A, C

E

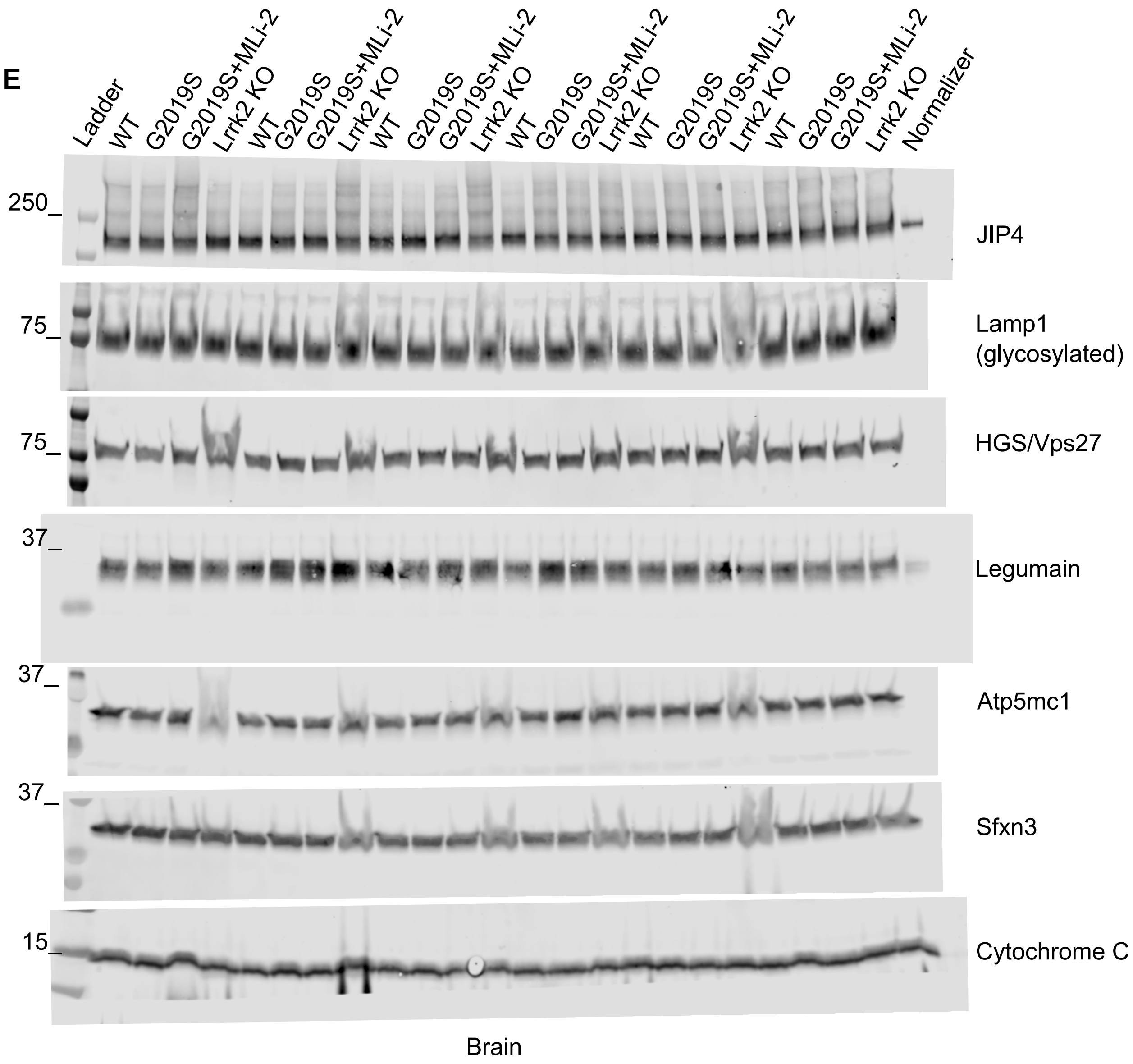
Ladder
WT
G2019S
G2019S+MLi-2
Lrrk2 KO
WT
G2019S
G2019S+MLi-2
Lrrk2 KO
WT
G2019S
G2019S+MLi-2
Lrrk2 KO
WT
G2019S
G2019S+MLi-2
Lrrk2 KO
WT
G2019S
G2019S+MLi-2
Lrrk2 KO
WT
G2019S
G2019S+MLi-2
Lrrk2 KO
Normalizer
250_
JIP4
75_
Lamp1
(glycosylated)
75_
HGS/Vps27
37_
Legumain
37_
Atp5mc1
37_
Sfxn3
15_
Cytochrome C
Brain

Fig. S6E

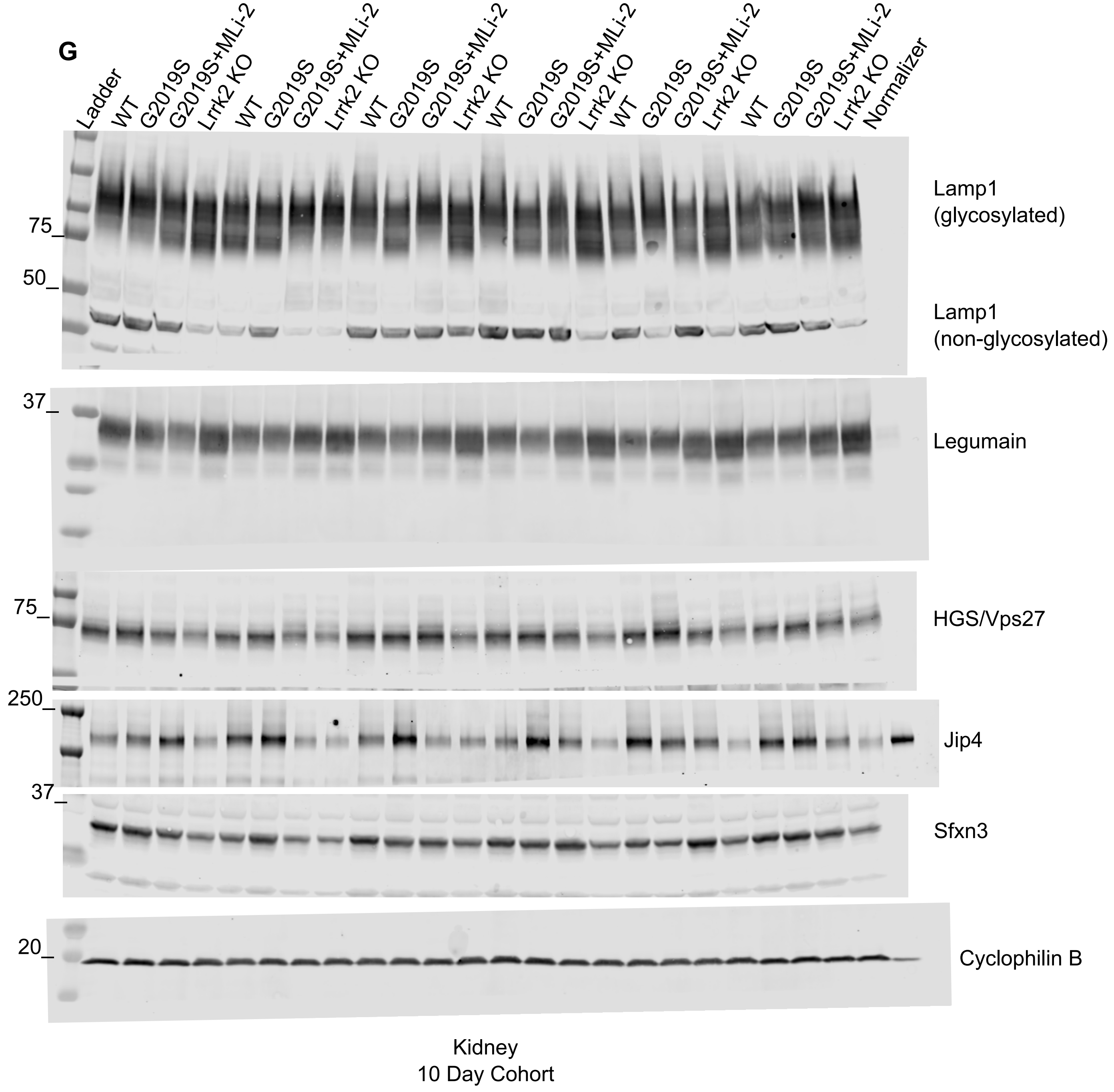
G
Ladder
WT
G2019S
G2019S+MLi-2
Lrrk2 KO
WT
G2019S
G2019S+MLi-2
Lrrk2 KO
WT
G2019S
G2019S+MLi-2
Lrrk2 KO
WT
G2019S
G2019S+MLi-2
Lrrk2 KO
WT
G2019S
G2019S+MLi-2
Lrrk2 KO
WT
G2019S
G2019S+MLi-2
Lrrk2 KO
Normalizer
75_
50_
Lamp1 (glycosylated)
Lamp1 (non-glycosylated)
37_
Legumain
75_
HGS/Vps27
250_
Jip4
37_
Sfxn3
20_
Cyclophilin B
Kidney
10 Day Cohort

Fig. S6G
